# Near-Infrared and Sono-Enhanced Photodynamic Therapy of Prostate Cancer Cells Using Phyto-Second Harmonic Generation Nanoconjugates

**DOI:** 10.3390/polym17131831

**Published:** 2025-06-30

**Authors:** Efrat Hochma, Michael A. Firer, Refael Minnes

**Affiliations:** 1 Department of Physics, Ariel University, Ariel 4070000, Israel; hochmaefrat@gmail.com; 2 Department of Chemical Engineering, Ariel University, Ariel 4070000, Israel; 3 Adelson School of Medicine, Ariel University, Ariel 4070000, Israel

**Keywords:** nanoconjugates, second harmonic generation, two-photon absorption, photodynamic and sonodynamic therapy, Perovskite crystals, barium titanate, phytochemicals, extracts, rhein, cancer cells, reactive oxygen species, polarization, fundamental frequency wave, harmonic wave, dipole moment, argon and femtosecond lasers, DPBF, dielectrics, ferroelectrics

## Abstract

This study investigates near-infrared (NIR)-induced, Phyto-enhanced, second harmonic generation-mediated photodynamic therapy (Phyto-SHG-PDT) using barium titanate (BT)/rhein/polyethylene glycol 100 (PEG100) and BT/Yemenite “Etrog” leaf extract/PEG100 nanoconjugates. We compare continuous-wave (CW), multi-line Argon-ion laser illumination in the NIR range with high-peak-power femtosecond (fs) 800 nm pulses. Under CW NIR light, BT/rhein nanoconjugates reduced PC3 prostate cancer cell viability by 18% versus non-irradiated controls (*p* < 0.05), while BT/extract nanoconjugates exhibited 15% dark toxicity. The observed SHG signal matched theoretical predictions and previous CW laser studies. Reactive Oxygen Species (ROS) scavenger 1,3-diphenyl-isobenzofuran (DPBF) showed reduced absorbance at 410 nm upon NIR illumination, indirectly supporting SHG emission at 400 nm from nanoconjugates. Under fs-pulsed laser exposure, pronounced two-photon absorption (TPA) and SHG effects were observed in both nanoconjugate types. Our results demonstrate the effectiveness of BT/rhein nanoconjugates under both laser conditions, while the BT/extract nanoconjugates benefited from high-power pulsed excitation. These results highlight the potential of BT-based Phyto-SHG-PDT nanoconjugates for NIR and blue light applications, leveraging nonlinear optical effects for advanced photochemical cancer therapies.

## 1. Introduction

Cytotoxicity resistance emerged as a global public health concern in the late 1940s [1,2,3,4,5,6]. Similarly, tumor cells often develop resistance to drug therapies through genomic instability, becoming increasingly aggressive and difficult to treat [5,6]. Prostate cancer, which primarily affects men around the age of 65, remains one of the leading causes of cancer-related death [7,8,9,10,11,12,13,14,15,16]. PC3 prostate cancer cells, a model for bone metastasis, are particularly challenging to manage [7,8,9,10,11,12,13,14,15,16]. Traditional treatments, such as chemotherapy, radiotherapy, and surgery, are associated with significant side effects and often yield suboptimal outcomes [7,8,9,10,11,12,13,14,15,16]. This emphasizes the need for alternative treatments that are both effective and minimize side effects, while also requiring low energy consumption.

**Scheme A1:**
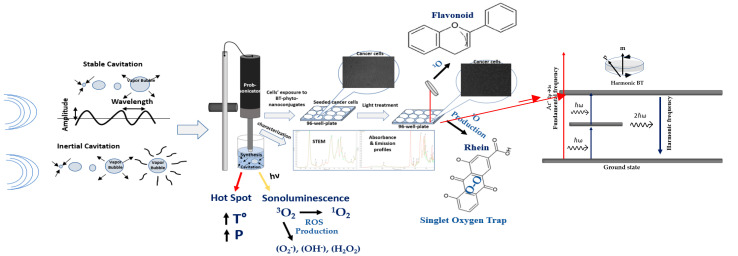
A schematic presentation of the low-frequency probe-sonication method for synthesizing BT-Phyto-nanoconjugates (left), as well as their characterization by STEM imaging (illustrative visualization), absorbance and fluorescence spectroscopy (center) and subsequent application to PC3 cancer cells via dark and NIR photo-toxicity investigation (right), resulting in ROS generation. The arrow indicates the progression from synthesis to characterization and application. Upon NIR illumination using an argon-based excitation source, BT nanoparticles exhibit SHG through both magnetic and electric resonances, enabling the activation of conjugated phyto-photosensitizers. Simultaneously, ultrasound during synthesis may initiate ROS generation via sonodynamic mechanisms (prob-sonication), including acoustic cavitation, sonoluminescence, and “hot spot” effects. Together, these pathways contribute to effective extracellular ROS production and cytotoxicity.

One promising strategy is to use natural and edible phytochemicals as cytotoxic agents and/or phyto-photosensitizers (PSs) to mediate physical treatments, such as photodynamic therapy (PDT), where cancer cells appear less likely to develop resistance [1,17,18,19]. Phytochemicals, which are bioactive molecules derived from plants, form part of the plant defense system and possess unique optical properties [7,9,17,18,19]. Many phytochemicals (particularly flavonoids and polyphenols) have diverse absorbance and emission spectra, making them suitable for physical cancer treatments, such as PDT and sonodynamic therapy (SDT) [1,2,7,9,17,18,19,20,21]. Furthermore, a more advanced approach involves conjugating these phytochemicals with nonlinear optical nanoparticles (see Scheme A1 in Appendix A) to enable blue light excitation via second harmonic generation (SHG) upon near-infrared (NIR) illumination [22,23,24,25], offering an improved and efficient treatment modality.

SHG, or frequency doubling, is a nonlinear optical process where two photons of identical frequency interact in a material with second-order susceptibility (χ^2^), generating a photon with twice the energy via coherent scattering (Scheme A1) [22,23,24,25,26,27,28,29,30,31,32,33,34,35].

Efficient energy transfer relies on the proximity between the non-centrosymmetric nanostructure and the PS. SHG nanoparticle illumination reveals size-dependent dynamics, with larger particles dominated by bulk effects and smaller ones by surface effects [22,27,28,36]. If the surface plasmon resonance (SPR) near-field effects align with the PS absorption maxima, the probability of an enhanced SHG effect, and, thus, increased ROS production via the PDT mechanism, is elevated, leading to cell destruction and death [23,26,27,28,36,37,38].

Dielectric nanostructures present a promising option to perform SHG-PDT due to two main advantages: the ability to support both magnetic and electric resonance excitations [26,28,39,40]. Magnetic resonances arise from circular loop currents induced within the particle by an incident light source (Scheme A1 and Equation (1)), characterized by a large mode volume that significantly enhances the SHG effect. Simultaneously, electric resonances are driven by oscillations of polarization charges (Scheme A1 and Equation (2)). This dual resonance capability of the magnetic and electric dipole moments (Scheme A1) makes dielectric nanostructures superior to metallic and photonic crystal ones for achieving efficient SHG [26,28,29,40]:

m (ω + 2ω + 3ω + …) = Ɛ_m_^(1)^b^(1)^E^(1)^ + Ɛ_m_^(2)^b^(2)^E^(2)^ + Ɛ_m_^(3)^b^(3)^E^(3)^ + … (1)

I = P (ω + 2ω + 3ω + …) = Ɛ_0_^(1)^x^(1)^E^(1)^ + Ɛ_0_^(2)^x^(2)^E^(2)^ + Ɛ_0_^(3)^x^(3)^E^(3)^ + … (2)

where **ω**, **Ɛ_0_**, **Ɛ_m_**_,_ **x**, **b**, **E**, **P**, and **m** are the fundamental frequency, the permittivity of free space and that of the material, the material’s susceptibility, the magnetic coefficient, the external electric field, the polarization or total power induced, and the magnetic moment induced, respectively.

PDT utilizes a PS to generate ROS in the presence of visible light (or NIR) and oxy-gen, inducing oxidative damage and cell death in cancer cells [1,22,23,41,42,43,44,45,46]. However, PDT is limited by the need for PSs with high absorption coefficients and by the restricted light penetration depth, particularly in deeper or larger tumors [22,23,41,42]. These challenges highlight the need for additional strategies to enhance traditional PDT. In this study, we hypothesize that BT/Phyto-PS nanoconjugates can enhance both conventional PDT and SHG-PDT by enabling efficient ROS generation from blue light-absorbing-PSs under NIR excitation, while supporting deeper tissue penetration. Unlike endogenous nonlinear structures within cells, these nanoconjugates offer an improved light-conversion platform for effective NIR-triggered phototherapy. To address the aforementioned limitations of PDT and ensure safe, effective deep cytotoxic therapy with reduced side effects and lower energy consumption, a combined approach using NIR-induced Phyto-SHG-PDT is proposed. This approach utilizes NIR lasers for collimated deep tissue penetration and blue light PS excitation, producing ROS to effectively target cancer cells while protecting surrounding healthy tissues. This is further supported by using natural Phyto-PSs, which exhibit low toxicity in non-cancerous cells [22,23,37,41].

In our previous studies, we developed effective nanoconjugation systems of perovskite barium titanate (BT) nanoparticles, known for SHG, with phytochemicals for SHG-mediated PDT, using Polyethylene Glycol (PEG100) as a linker, which provided colloidal stabilization and enhanced the interaction distance [22,23,45]. Additionally, we demonstrated that phytochemicals, including Yemenite “Etrog” leaf extracts and rhein, generate ROS under blue light excitation [37,47]. This study investigated conjugation between BT and these phytochemicals for NIR-induced SHG-PDT [22,23,37]. BT’s ferroelectric, non-centrosymmetric structure gives it significant intrinsic second-order nonlinear susceptibility, making it ideal for SHG applications [22,23,26,27,38,39]. The BT/PS nanoconjugates were synthesized via low-frequency (40 kHz) prob-sonication (Scheme A1), with ultrasound promoting SHG through phase transitions and symmetry breakage [1,2,20,21,26,39]. Nanocomposites were evaluated using Scanning Transmission Electron Microscopy (STEM) and spectral analysis. The nanoconjugates were applied to PC3 cancer cells, with cell viability assessed under dark conditions and NIR laser treatment (Scheme A1). SHG signal analysis from BT aligned with theoretical predictions and prior studies for CW lasers, supporting SHG detection. Singlet oxygen production, investigated using DPBF, showed reduced absorbance at 410 nm upon NIR illumination, linking SHG emission to ROS production. The absorption spectra of BT + DPBF, DPBF + BT/PS nanoconjugates, and DPBF + PS mixtures were analyzed for varying illumination times.

Finally, a NIR 800 nm high-peak-power femtosecond pulse laser illumination was employed to compare the emission spectra generated by the PSs and BT/PS nanoconjugates under 800 nm (two-photon, both real and virtual) and 400 nm (one-photon) excitations. The interaction of closely spaced NIR multi-line emissions from a CW Argon-ion laser with nonlinear BT nanoparticles and their BT/Phyto-PS nanoconjugates (with matching bandgaps for both BT and rhein PS), applied to cancer cells, offered a distinct approach for achieving SHG with simple laser setups. BT/Phyto-PS-based nanoconjugates are proposed as cytotoxic agents and/or photo-mediators for NIR-induced SHG-PDT for medical applications.

## 2. Materials and Methods

### 2.1. Phyto-Photosensitizer Stock Preparation

Rhein was purchased from Angene (Hong Kong) and dissolved in absolute ethanol to prepare a 0.1 mg/mL stock solution, assisting ultrasound sonication for 30 min in total (three cycles of 10 min in an ultrasonic bath with ice water replacement) to achieve a uniform solution. Yemenite citrus medica (“Etrog”) extraction was prepared as described in our previous paper [37]. In brief, the leaves were collected in September 2023 from Ariel city, Israel. The leaves were washed, dried at 80 °C for 4 h, and ground into a fine powder. This powder was then incubated overnight in absolute ethanol (1:10 ratio) while stirring. After filtration, the extract was concentrated to 3.5 ± 0.35 mg/mL using a rotary evaporator.

### 2.2. Synthesis of BT-Phyto-Photosensitizer Nanocomposites

A colloidal suspension of the Phyto-SHG-nanocomposites was synthesized by mixing and sonicating 6 mg of perovskite BT nanoparticles (467,634, Sigma-Aldrich, St. Louis, MO, USA) in double-distilled water (DDW) (≤0.5 µs/cm) as the main phase, along with a 6% *v*/*v* PEG100 (mixture of DPPC and PE-PEG) and a 0.33% *v*/*v* photosensitizer in the case of rhein (0.1 mg/mL stock solution, 1,8-Dihydroxy-3-carboxyanthraquinone, 478-43-3, AG0037FB, Angene), as well as 2.66% *v*/*v* extract (3.5 ± 0.35 mg/mL stock solution). This resulted in a final volume of 15 mL, with a BT concentration of 0.4 mg/mL and BT/rhein and BT/extract PDT particle concentrations of 400.3 µg/mL, and 493 µg/mL, respectively. Sonication (BW-150 model) was applied for 15 min with a maximum input power of 120 W, a frequency of 40 kHz, an 80% amplitude, and 1 sec on/4 s off pulse irradiation at 4 °C (Scheme A1). The phyto-nanocomposites were then filtered through a 0.22 µm PVDF membrane filter before undergoing PDT mediated by SHG treatment.

### 2.3. Scanning Transmission Electron Microscopy (STEM) Imaging of BT/Phyto-Nanoconjugates

The morphology and size distribution of BT and BT-based phyto-nanoconjugates were investigated and examined using high-resolution scanning electron microscopy (Ultra-High-Resolution Maia 3 FE-SEM, Tescan, Brno, Czech Republic) with a STEM (bright and dark) detector and an electron beam voltage of 25 kV. A 10 µL aliquot of the diluted sample (Section 2.2) was placed on a silicon wafer grid and dried overnight. Particle size was estimated using ImageJ software (version 1.54m).

The nanoparticles exhibited a mean diameter of ~50 ± 8 nm and a tetragonal phase, as confirmed by high-resolution STEM imaging (Section 3.1), and an optical bandgap calculation of ~3.2 eV, as confirmed via UV–Vis analysis (Section 3.1), respectively—values consistent with the reported tetragonal BaTiO_3_ and favorable for SHG efficiency. The detailed bandgap calculation used was as follows:

(3) $$E\left(eV\right)=\frac{hc}{\lambda \;\left(nm\right)}$$

*BT*(*Bandgap*) (eV) = 1240/*BT*(*λ_absorption_max*) (nm) = 1240/385 nm = 3.22 eV

### 2.4. Photophysical Characterization

Composite productions were examined via absorbance and emission profile investigation of BT, RH, and EXT alone and in their nanoconjugation combinations: BT/RH/PEG100 and BT/EXT/PEG100. The absorbance spectrum analysis was performed at room temperature using a V-730 UV–Visible spectrophotometer (Jasco, Tokyo, Japan), 10 mm pathlength quartz cuvettes, and a scan rate of 1000 nm/min. The fluorescence spectrum analysis was performed with a FP-8500 spectrofluorometer (Jasco, Tokyo, Japan) under the following conditions: excitation at 400 nm, excitation and emission bandwidths of 5 nm each, a response time of 0.1 s, medium sensitivity, a 420–750 nm measurement range, a 1 nm data interval, and a 500 nm/min scan speed.

### 2.5. Cancer Cell Line Growth

Human prostate cancer PC3 cells (ATCC-CRL-1435) were cultured in RPMI-1640 medium at 37 °C with 5% CO_2_, supplemented with 10% heat-inactivated FBS, 100 U/mL of penicillin, 100 µg/mL of streptomycin, and 5% of L-glutamine. Cells were grown in T-25 Falcon flasks until 80% confluency, then detached, centrifuged, and resuspended in fresh medium. Cell counting was performed using a TC 20TM Automated Cell Counter with a 1:1 ratio of cells to trypan blue. Finally, cells were seeded into a 96-well microplate at a density of 2 × 10^5^ cells/mL. The PC3 cell line was obtained from the American Type Culture Collection (ATCC), located in Manassas, VA, USA.

### 2.6. Dark and NIR-Induced Phyto-SHG-PDT Treatments Against Prostate Cancer Cells

Cytotoxicity and cyto-phototoxicity analyses were conducted as described in our previous paper [37], albeit with some changes. Adherent prostate PC3 cancer cells (100 µL, 2 × 10^5^ cells/mL) were seeded in a 96-well plate (Nunclon, Thermo Fisher Scientific, Waltham, MA, USA) and incubated overnight to reach 80% confluency. The BT-Phyto-PS nanocomposites (see Section 2.2) were introduced directly to the cells for a 1 h pre-incubation period, guided by fluorescence emission intensity readings (refer to Section 2.6 in [37]), followed by a 30 min argon-based NIR light treatment (see Section 2.7 and Scheme A1 in Appendix A). After 24 h, an XTT cell viability assay was performed: the medium was removed and replaced with 100 µL of fresh medium, followed by the addition of 50 µL of activated XTT solution according to the manufacturer’s instructions (cell viability assay, BI Biological Industries Catalog No. 20-300-1000). The XTT assay is a colorimetric method that measures mitochondrial enzyme activity as an indicator of cell viability. Absorbance was measured at 450 nm (with a 580 nm reference wavelength) using a microplate reader to determine cell viability. Dark toxicity was assessed in parallel plates under the same conditions. Control wells containing only cells were included in the measurements.

### 2.7. Cytotoxicity of CW Argon-Ion-Based Laser

**Scheme A2:**
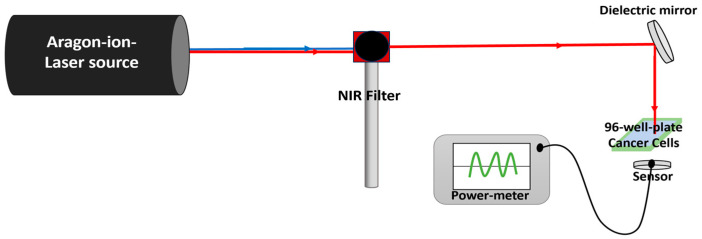
A schematic representation of an NIR-induced Cyto-SHG-PDT system.

Laser toxicity analysis was conducted mostly as described in our previous paper [37], albeit with a few modifications. In brief, human prostate PC3 cancer cells (100 µL, 2 × 10^5^ cells/mL) were seeded in a 96-well plate and incubated overnight to reach 80% confluency. The cells were then illuminated in the NIR with an argon-ion laser (1 mm diameter, closely spaced argon multi-emission lines in the range of 696 nm to 968 nm [48,49,50,51], 25 mW/cm^2^ average power density). More specifically, the laser light source was passed through a NIR bandpass filter (700 nm–1100 nm), reflected at a 45° angle by a dielectric mirror, and vertically focused onto the 96-well plate containing the cells (Scheme A2 in Appendix A). Cells were illuminated, leading to a reduction in cell viability of no more than 10% due to laser-induced toxicity, maintaining 90% cell viability.

### 2.8. ROS Generation Investigation in BT Nanoparticles and BT-Phyto-PS Nanocomposites Under NIR Continuous Argon Laser Illumination

**Figure A1:**
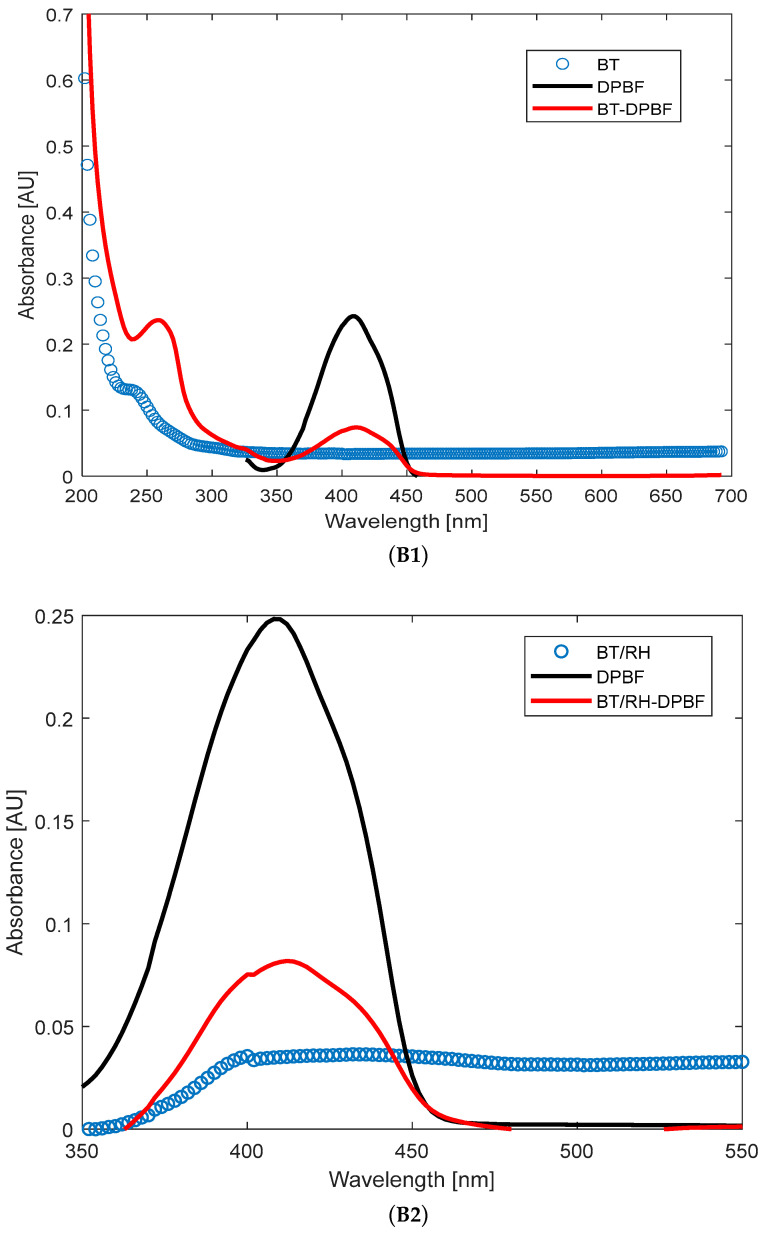

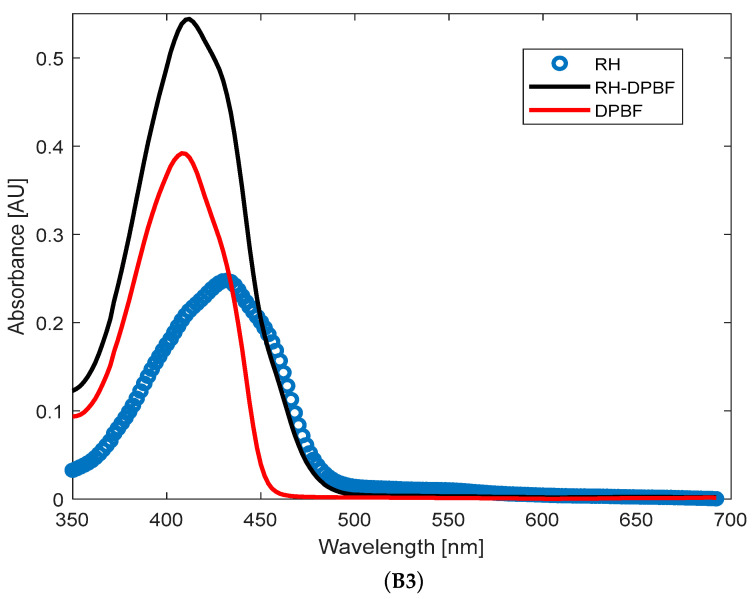
An investigation of ROS production process by BT/rhein nanoconjugates and their individual components (BT and rhein). (**B1**) The absorbance levels of BT and the singlet oxygen indicator DPBF at 410 nm at time zero (before NIR illumination). The absorbance of their mixture (BT + DPBF) at 410 nm at time zero is also presented. (**B2**,**B3**) absorbance levels of BT/rhein (BT/RH) and rhein (RH) relative to the singlet oxygen indicator DPBF at 410 nm at time zero (before illumination). The absorbance of their mixture (BT/rhein + DPBF and rhein + DPBF) at 410 nm at time zero is also presented.

The production of singlet oxygen by BT, RH, and their nanocomposite BT/RH/PEG100 was investigated and evaluated using the photochemical probe 1,3-diphenyl-isobenzofuran (DPBF) [37]. DPBF was dissolved in methanol to achieve an absorption of 0.25 at 410 nm (measured using a UV–Vis spectrophotometer, Jasco, Tokyo, Japan, with a 1 cm path length, in arbitrary units) for a final volume of 3 mL, then combined with the BT and BT/RH/PEG100 (2 mL each, with concentrations of 0.4 mg/mL for BT and 0.4 μg/mL for RH in the nanoconjugate; see Section 2.1 and Section 2.2) and Appendix B). This resulted in an absorbance of 0.035 at 410 nm for the samples (Figure A1(B1,B2) in Appendix B). For the RH + DPBF mixture, the absorbance levels were 0.25 (RH) and 0.4 (DPBF), Figure A1(B3). These absorbance levels at time zero (before illumination) ensured that DPBF was in excess, allowing for full singlet oxygen trapping and preventing aggregation bands. It should be mentioned that DPBF has an absorption maximum band at 410 nm, and the efficiency of singlet oxygen trapping was indicated by the reduction in this band over the illumination period. Control experiments were conducted with only BT and only DPBF (compared to BT + DPBF and BT/RH/PEG100 + DPBF) under the same illumination conditions over time. These controls were essential to confirm that the decrease in absorbance at 410 nm was solely due to DPBF reacting with singlet oxygen produced by the investigated analytes (BT and BT/RH/PEG100) under laser illumination. RH alone was not included as a control since the RH + DPBF sample did not show any ROS production under continuous NIR argon-based laser illumination (Section 2.7 and Section 2.9). A plot showing the natural logarithm of normalized DPBF absorbance at 410 nm against time was generated to calculate the singlet oxygen production rate constants (k) from the slope of the fitted pseudo-first-order reaction:

ln(N/N_0_) = −kt (4)

where N is the DPBF concentration at 410 nm over time, N_0_ is the initial concentration of DPBF, and k is the degradation rate constant of DPBF [min^−1^].

### 2.9. SHG Investigation from BT Nanoparticles and BT-Phyto-PS-Nanocomposites Using Continuous NIR Argon-Ion-Based Laser Illumination

**Scheme A3:**
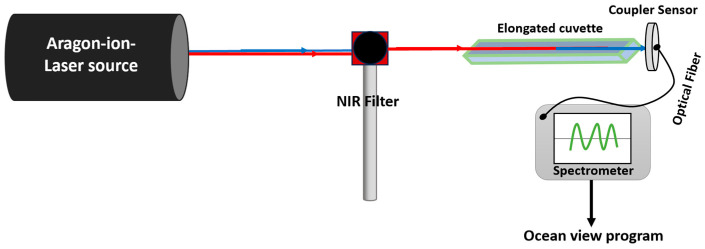
A schematic representation of an SHG-signal detection system.

Samples of BT, RH, BT/RH/PEG100, and BT/EXT/PEG100 were excited using an argon-ion laser beam passed through a NIR bandpass filter. To enhance light–matter interaction, the samples were placed in elongated quartz cuvettes to increase the optical path length. Potential SHG emission signals were recorded in real time using an Ocean Optics QE Pro Spectrometer (Ocean Insight, Orlando, FL, USA); see Scheme A3 in Appendix A and Section 2.7 for more details. The application of NIR induced SHG-PDT treatment on prostate cancer cells using the argon-based laser was evaluated through a cell viability assay (see Section 2.6). The samples were also excited by the argon-based laser in DPBF singlet oxygen probe mixtures (BT + DPBF, RH + DPBF, and BT/RH + DPBF) to investigate ROS production (Section 2.8). DPBF alone was also illuminated with the argon-based laser as an additional control. Furthermore, the BT nanoparticle samples were excited after the laser beam passed through polarizers set at different angles and varying power levels (see Section 2.11).

### 2.10. SHG Investigation from BT Nanoparticles and BT-Phyto-PS-Nanocomposites Using NIR High-Peak-Power Femtoseconds Pulse Laser Illumination

The Phyto-SHG-nanocomposite samples (BT/Phyto-Photosensitizer/PEG100, see Section 2.2) and their controls (BT nanoparticles and Phyto-Photosensitizer nano-dyes, Section 2.1 and Section 2.2) were excited by a NIR fs pulse laser light source (emission wavelength of 800 nm, average power density of 300 mW/cm^2^, pulse length of 35 fs, and repetition rate of 71.4 MHz), and the emission signals were recorded in real time using an Ocean Optics QE Pro Spectrometer (Ocean Insight, Orlando, FL, USA). Alignment of the beamline was achieved using dielectric mirrors.

### 2.11. Polarization and Power-Controlled Investigation of NIR Argon-Ion-Based Laser Interactions in BT Nanoparticle Suspensions

**Scheme A4:**
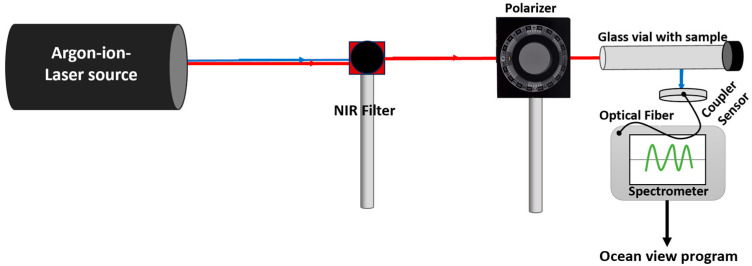
A schematic representation of a polarization-controlled NIR excitation system.

The BT + DPBF samples were illuminated with closely spaced multi-line NIR light, passed through a NIR bandpass filter and polarizers set at 30° and 70° relative to the p-polarized incident beam, with a control sample illuminated without a polarizer. All samples were placed in 1.5 mL transparent glass vials to ensure uniform light exposure and consistent optical geometry (Scheme A4 in Appendix A and Section 2.6, Section 2.7, Section 2.8 and Section 2.9). Singlet oxygen production efficiency was assessed by monitoring the decrease in DPBF’s absorbance at 410 nm (Section 2.8). For power dependence experiments, the singlet oxygen production rate constants (k) of BT + DPBF samples were investigated under different NIR illumination powers (0, 0.037 mW, and 25 mW) and analyzed for trends (Supplementary to 3.3).

### 2.12. Statistics

Each measurement was conducted in at least two independent experiments, with cell viability assays performed in well triplicate to ensure reproducibility (variability minimization). In experiments involving the photochemical probe DPBF, measurements were taken over multiple time points to track photodegradation, with each data point being measured at least four times across the experimental timeline, ensuring temporal consistency and accuracy in photodegradation tracking. The results obtained were analyzed by a *t*-test (unpaired two-tailed Student *t*-tests). A statistical significance threshold was applied, considering results to be significant when the *p*-value was below 0.05 (** *p* < 0.05). Data are expressed as mean values ± standard deviation (SD) to reflect the variation in and consistency of the measurements.

## 3. Results and Discussion

This study investigated the nonlinear optical properties of perovskite BT nanoparticles for potential conjugation with blue-visible light-absorbing natural, plant-derived photosensitizers, namely rhein (RH) molecules and Yemenite Citron leaf extracts (EXTs). The objective was to assess the performance of these proposed ferroelectric nano-systems in facilitating the excitation of phyto-PSs through blue light emission generated by the SHG effect while simultaneously achieving deep tissue penetration via NIR illumination. In other words, the goal was to achieve a detectable SHG fluorescence signal at 400 nm, originating from the harmonic BT nanoparticles upon NIR illumination. This method permits the use of a sufficiently long illumination wavelength for deep tissue penetration coupled with utilizing a shorter wavelength to generate a significant amount of ROS. To achieve this goal, we first investigated and evaluated the synthesis and formation of the Phyto-SHG nanoconjugates using STEM imaging and UV–Vis absorbance and emission spectra analysis. Next, we exposed the cells to the BT/Phyto-nanoconjugates both in the dark and under NIR light treatment, demonstrating their impact on cell viability. For mechanism investigation, the SHG signal from BT was observed, consistent with the experimental setup and previous studies using CW lasers. Additionally, we employed the ROS indicator DPBF, with an absorption maximum at 410 nm, to confirm ROS generation and compare the ROS production efficiency of the nanoconjugates with their individual components, further supporting the SHG findings at 400 nm. Using both CW and pulsed lasers, a direct and real-time method for quantifying ROS generation through SHG signals was achieved, offering a new pathway for ROS measurement. The impact of illumination with a high-peak-power femtosecond 800 nm pulsed laser on the nanoconjugated system was also demonstrated. Finally, we proposed a prominent light–matter interaction mechanism, with the overall investigation steps outlined in the following sections.

### 3.1. Synthesis and Characterization of Phyto-SHG Nanoconjugates: Structural and Spectral Insights

The synthesis and formation of the BT-Phyto-SHG nanoconjugates were initially investigated and evaluated through Scanning Transmission Electron Microscopy (STEM) imaging, as well as UV–Vis absorption and emission spectral analysis.

**Figure 1:**
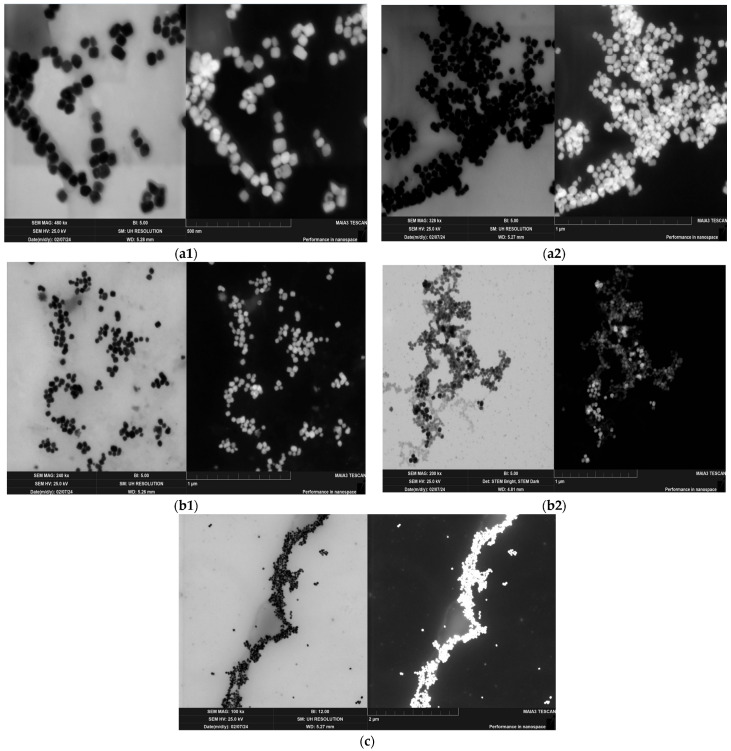
Bright- and dark-field STEM images of barium titanate (BT)/PEG100 and BT nanoconjugates at different magnifications and configurations: (**a1**,**a2**) BT/rhein/PEG100, (**b1**,**b2**) BT/Yemenite “*Etrog*” leaf extract/PEG100, and (**c**) bare BT/PEG100 after probe sonication synthesis. Electron beam voltage: 25 kV; magnification: 100–480 kx; scale bar: 500 nm (**a1**), 1–2 µm (**a2**,**b1**,**b2**,**c**).

STEM analysis revealed that the BT/RH/PEG100 (Figure 1(a1,a2)) and BT/EXT/PEG100 (Figure 1(b1,b2)) nanoconjugates exhibited round PEGylated nanoparticles with diameters of 51 ± 23 nm and 48.6 ± 7.5 nm, respectively. The nanoparticles displayed low size distribution and high uniformity in shape, ensuring that the desired nonlinear optical response would be uniform and consistent across different crystal orientations. Additionally, a thin organic film coating the BT nanoparticles was observed in both the RH and EXT nanoconjugates, where, in the case of the extract, the organic coating was more dominant and widespread (Figure 1 (a1,a2) VS. (b1,b2)). This uniformity and coating are essential for enhancing the optical properties of the nanoconjugates. Notably, the greater distance observed between nanoparticles in the extract-based nanoconjugates (relative to rhein-based and BT-alone samples) visually supports our proposed stability hierarchy: extract nanoconjugates > rhein nanoconjugates > BT. This spatial arrangement, which stems from the extract’s molecular composition, contributes to enhanced colloidal stabilization and is directly observable in the presented image (Figure 1(b1,b2)). Additionally, Figure 1(a1,a2, and b1) (magnifications of 480 kx, 328 kx, and 240 kx, respectively) offers enhanced resolution at higher magnifications, effectively highlighting the distinct morphology of individual nanoparticles and revealing a uniform distribution within the nanoconjugates. These images show a more dispersed nanoconjugate arrangement, with visibly separated particles and blurred edges, probably due to tip sonication during dispersion. The increased contrast between the lighter peripheral layer and the denser inorganic core supports the presence of an organic coating. In contrast, Figure 1c (100 kx magnification) illustrates longer chains of nanoparticles, emphasizing the impact of surface interactions and surface effects in bare BT samples. The morphological differences between the coated nanoconjugates and solely BT nanoparticles samples are thus clearly distinguishable.

**Figure 2:**
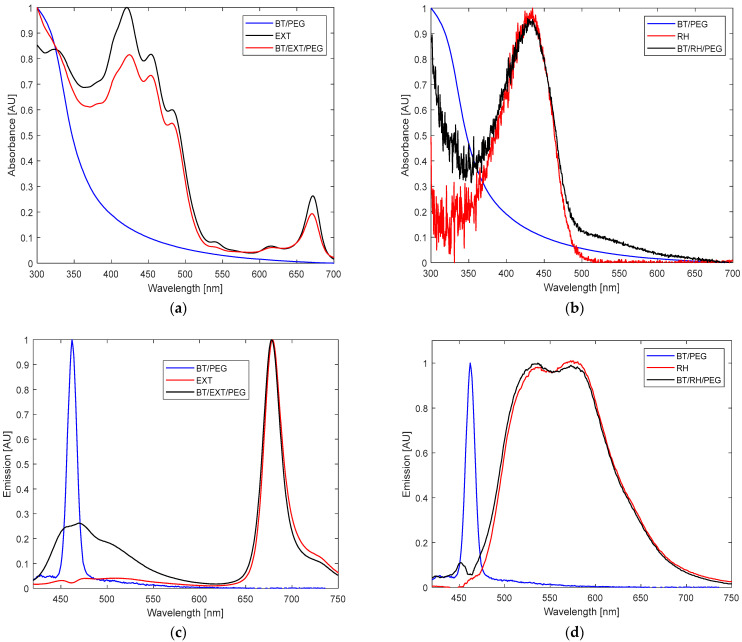
(**a**,**b**) The absorbance (in the range of 300 nm to 800 nm) and (**c**,**d**) fluorescence (420 nm to 750 nm; 400 nm excitation; slit: 5-5; sensitivity: medium) spectra of BT/PEG (barium titanate with PEG coating), Yemenite “Etrog” leaf extract (EXT), BT/EXT/PEG (barium titanate conjugated with EXT), rhein (RH), and BT/RH/PEG (barium titanate conjugated with rhein) in double-distilled water (DDW).

The UV–Vis absorbance spectrum of EXT alone showed a maximum at 330 nm; a shoulder at 392 nm; three dominant peaks at 420, 455, and 484 nm; smaller signals at 535 and 620 nm; and an additional peak at 675 nm (Figure 2a). In contrast, BT alone showed no significant absorbance signal between 350 and 400 nm, but it instead displayed an elongated, gradual rise between 300 and 400 nm, a trend also observed in [22,23,38] (Figure 2a). The combined BT/EXT/PEG100 samples exhibited a pronounced bleaching effect accompanied by a red shift in the visible spectrum, along with a coil-elongated pattern, indicating successful conjugation formation (Figure 2a). Similarly, the BT/RH/PEG100 nanocomposites displayed a marked spectral inflection between 300–400 nm, absent in RH alone, which showed a single absorbance peak centered at 433 nm (Figure 2b).The fluorescence emission spectra upon 400 nm excitation exhibited a narrow peak at 675 nm for EXT and a broad, split signal between 470 and 700 nm for RH, with maxima at 530 and 580 nm (Figure 2c vs. Figure 2d). The emission profiles of BT/EXT/PEG100 and BT/RH/PEG100 showed quenching effects. Additionally, in the BT/EXT/PEG100 sample, the signal associated with BT exhibited a significant amplitude at 463 nm, aligning with the emission signal of BT alone (Figure 2c). On the other hand, the BT/RH/PEG100 spectrum demonstrated an overall blue shift, with the BT-related signal shifting towards 453 nm compared to the original 463 nm signal, alongside a reduction in amplitude (Figure 2d). The absorbance–fluorescence observations suggest potential interactions within the nanoconjugates, with an increased probability of energy transfer between BT and RH through conjugate formation.

Extracts from the peels, pulp, juice, and leaves of closely related varieties have been found to contain compounds characterized by extensive conjugated systems of aromatic rings, particularly flavonoids and polyphenols, which are the primary secondary metabolites in these extracts [7,9,17,18,19,20,21,51,52,53,54,55]. According to studies [56,57], coupling amino acids or peptides with PEG can significantly enhance their conjugation potential. The intrinsic properties of the extract, containing both hydrophilic and hydrophobic molecules [37], align well with the hydrophobic environment of PEG100 phospholipids, which may explain the robust BT/EXT/PEG100 conjugation observed. Furthermore, rhein, a small amphiphilic molecule (supports interaction with PEG100) with a molecular weight of 284.22 g/mol, exhibits acidic properties and can contribute protons (H^+^) in aqueous media, resulting in a negative charge. This negatively charged state induces electrostatic interactions with the primary surface hydroxyl groups on BaTiO_3_, facilitating strong adhesion. Hydrogen bonding between the rhein and BT functional groups is also possible. The observed shift in the signal representing BT within the BT/RH emission spectra (Figure 2d) strengthens the claim of a strong interaction in the BT/RH/PEG100 case. Moreover, considering the absorption coefficient of rhein (ε = 5 × 10^3^ Lmol^−1^ cm^−1^ at 400 nm) and comparing the absorption spectra of BT/RH with those of BT and rhein, the final concentration of rhein loaded onto BT can be calculated using the Beer–Lambert law: it is 80 µM, which falls well in the range of PS’s loading (1–100 µM) [22,23,37,47]. Next, we applied the nanoconjugates to the cells, as detailed in the following section (Section 3.2).

### 3.2. Dark and NIR-Induced SHG-PDT Activity of BT-Phyto-Nanoconjugates Against Prostate Cancer Cells

**Figure 3:**
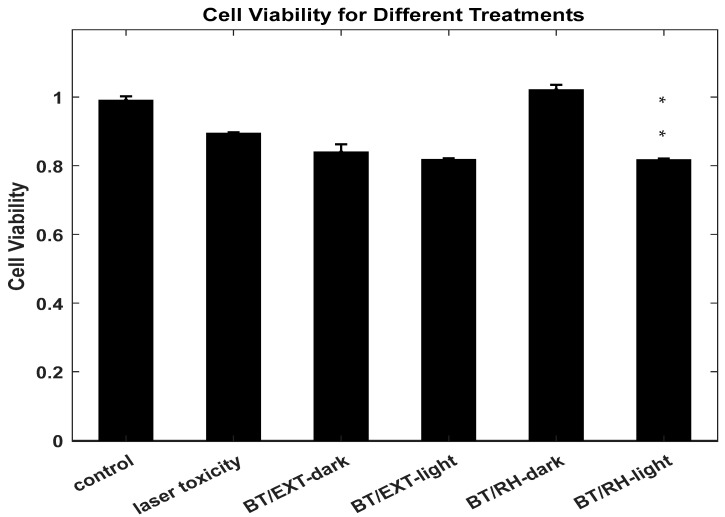
The cell viability assay of BT/rhein (BT/RH) and BT/extract (BT/EXT) nanoconjugates following a 1 h pre-incubation period, then 30 min of dark toxicity exposure or light treatment (dark parallel plate kept under the same conditions as the light treatment). Untreated cells and cells exposed to laser toxicity only were used as controls. NIR illumination was performed using an argon-ion-based laser (25 mW, 30 min). Cell survival assay *n* = 2 for independent experiments with well triplicates; BT/RH and BT/EXT PDT particle concentrations: 400.3 µg/mL and 493 µg/mL, respectively; *Y*-axis values were normalized to untreated controls, with 100% viability set to 1. Cell viability was calculated by subtracting the baseline absorbance from the sample absorbance and dividing by the baseline. Error bars (±SD) represent 1 standard deviation; *p*-value < 0.05 (**) was calculated by a *t*-test. Asterisks (**) indicate significance in the BT/RH-light treatment case.

**Scheme A5:**
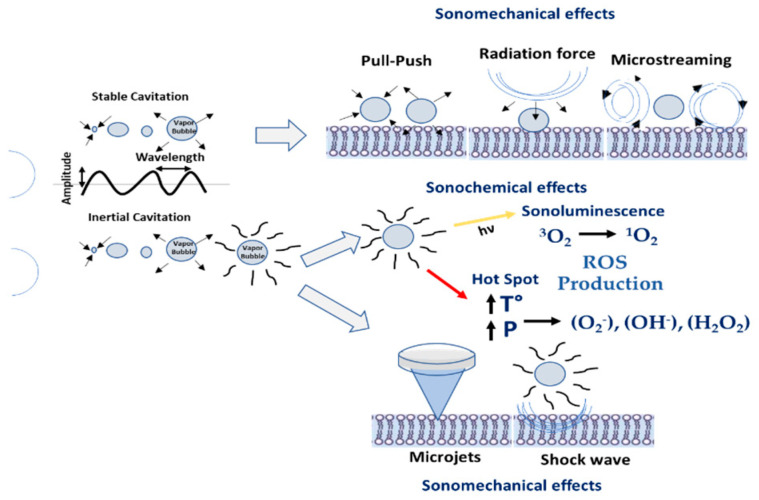
The sonodynamic cyto-deactivation mechanism (of both stable and inertial cavitation cases), including sonoluminescence, “hot spot”, and sonolysis phenomena leading to ROS production (inspired by [1]).

Based on our previous study [37], we applied optimized working parameters, including a BT-Phyto-nanoconjugate concentration and a dark pre-incubation time of 1 h (see also Section 2.1, Section 2.2, Section 2.6 and Section 2.7) to conduct NIR-induced Phyto-SHG-PDT treatment. The phyto-nanoconjugates were cultured with the cells in the dark for 1 h in a 96-well plate before being subjected to light treatment. The emission lines from an argon-ion-based laser pump source were filtered using a near-infrared bandpass filter and reflected by a dielectric mirror positioned at a 45° angle relative to the incident beam (Section 2.6). This setup ensured direct contact between the light beam and the 96-well plate containing the cells (Section 2.6). Illumination was applied for 30 min, and cell viability was assessed 24 h later using the XTT assay. Illumination with the laser’s NIR fundamental wavelengths alone (laser toxicity) led to a 9.67% reduction in cell survival compared to the control (Figure 3). The combined NIR-induced Phyto-SHG-PDT treatment mediated by BT/rhein nanoconjugates reduced cell viability by 18% compared to non-irradiated controls (** *p* < 0.05), which aligns well with previous studies of the BT/Ce6 system applied to cancer cells [25,47,57]. While this level of cytotoxicity may appear modest, it is significant in the context of our biocompatible and non-invasive SHG-PDT system, which uses CW NIR light to activate natural PSs. This outcome is consistent with previous findings using similar BT-based nanoplatforms and supports the feasibility of our approach as a basis for further optimization toward enhanced therapeutic efficacy. Fibrillar collagen within PC3 cancer cells is known to exhibit nonlinear optical effects [57], albeit with low efficiency. This justifies the use of our BT/Phyto-PS nanoconjugated system. On the other hand, under the same experimental conditions, the BT/extract nanoconjugates exhibited dark toxicity, resulting in a 15% reduction in cell viability (Figure 3). This outcome probably results from the insufficient proximity of the photo-active fraction within the extract to the harmonic BT nanoparticles, reducing the probability of Phyto-PS excitation. Furthermore, other phyto-constituents within the extract may cause screening between the BT nanoparticles and the photo-phyto-active fraction, leading to reduced excitation and, consequently, low ROS production upon light treatment. In addition, antioxidants in the extract, such as carotenoids, may actively quench singlet oxygen through chemical reactions, thereby reducing ROS availability. As a result, constituents in the extract may act not only as PS but also as protective antioxidants, especially under ultrasound treatment, serving as a shield that mitigates oxidative stress. Moreover, ROS generation may begin as early as the initial synthesis stage, during the ultrasound operation applied for nanoconjugation [1,2,21]. Sonosensitizers (SSs) such as rhein have been reported to continuously produce ROS once sono-activated [58], potentially contributing to the cancer cell eradication effort. Ultrasound, characterized by the transmission of longitudinal pressure waves through a medium at frequencies above 20 kHz (above the human hearing threshold), is also known as SDT [1,2,21,59,60,61,62,63,64,65,66,67,68]. In liquids, its mechanism involves acoustic cavitation—violent microbubble collapse—which induces sonomechanical and sonochemical effects (see Scheme A5 in Appendix A).

Under low-intensity ultrasound (frequencies typically between 1 MHz and 3 MHz), stable cavitation may occur, leading to microstreaming, radiation forces, and the mechanical shear stresses of push–pull effects.

In contrast, high-intensity ultrasound (frequencies between 20 kHz and 100 kHz) can cause inertial cavitation, producing shock waves and microjets (depending on bubble size), as well as sonoluminescence and “hot spot” phenomena (Scheme A5) [1,2,21,59,60,61,62,63,64,65,66,67,68]. These effects are directly associated with the excitation of SS through ultrasound activation, resulting in the production of ROS.

SS are also known to produce ROS through sonolysis, and under light excitation appearance as a result of microbubble collapse from the cavitation effect [1,2,21,59,60,61,62,63,64,65,66,67,68]. Hatanaka et al. [69] reported on a single-bubble sonoluminescence phenomenon in distilled water at 250–700 nm, where the highest light emission was achieved in the UV region between 300 and 350 nm. This information could also explain the extract nanoconjugate’s cytotoxicity performance. We previously demonstrated via ATR-FTIR [37] that the extract contains both polar (hydroxyl (O–H), amide A (N–H), and C=O carbonyl), and nonpolar (aliphatic (C–H), and terpenes-related bands) functional groups—signatures of phenols, proteins, fatty acids, and terpenes. Aromatic rings vibrations and polyphenols’ bands were also observed. These groups can promote ROS formation via auto-oxidation/redox cycling and Fenton-like reactions, even without light. Together, these biochemical characteristics my explain the observed ~15% dark cytotoxicity of the extract. In the case of rhein, the absence of dark toxicity is probably due to its concentration (and exposure time) in the aqueous medium, as rhein is known for its sonosensitizing capabilities [70,71,72]. Moreover, Zhang et al. and Li et al. [73,74] reported on the ability of lipophilic structures to perform self-assembly in the context of rhein’s sonodynamic performance. As a result of this probable self-assembly behavior during sonication, core encapsulation of ROS produced during the process could occur, potentially impairing the overall sonodynamic effect.

Building upon the findings of NIR-induced Phyto-SHG-PDT, we further investigated the mechanism of action by comparing the effects of CW argon-based laser illumination to femtosecond pulse laser exposure.

### 3.3. Mechanism Investigation of BT and BT-Phyto-Nanocomposites Interacting with a Continuous Argon-Based Laser Compared to Femtosecond Pulse Laser Ilumination

#### 3.3.1. Investigation of SHG in BT Nanoparticles Under NIR Continuous Argon Laser Illumination

To study and analyze the results obtained from applying the BT-Phyto-nanoconjugates to the cells, a comprehensive understanding of the CW argon-ion-based laser operation was essential (see Supplementary to 3.3.1). BT nanoparticles were studied and found to exhibit nonlinear optic effects under CW lasers, particularly SHG [22,23,38,75,76]. Previous studies have also observed TPA in BT crystals within the 400 nm to 700 nm range and in the 1500 nm region under pulsed laser conditions [77]. The TPA cross-section is an intrinsic material property, therefore, remains relevant in our study, especially under pulsed laser excitation. However, under CW laser conditions, our primary focus is on the SHG phenomenon.

**Figure 4:**
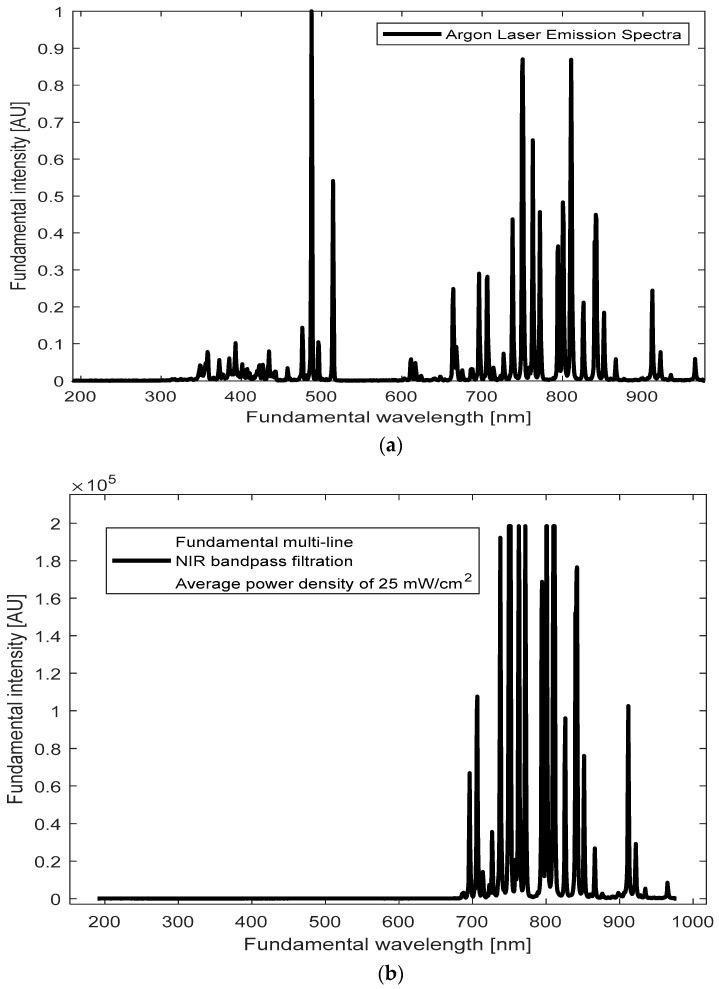

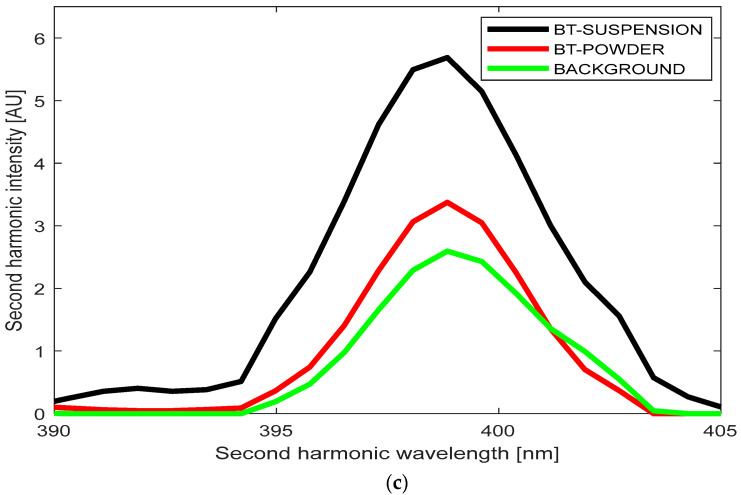
The emission spectra of (**a**,**b**) an argon-ion-based laser, featuring simultaneous and continuous spectral lines in the visible (400 nm to 514 nm) and near-infrared (696 nm to 968 nm) regions, respectively. The NIR region includes Ar^+^ 2p→1s transitions, where the closely spaced multi-emission lines provide an average power density of 25 mW/cm^2^. To isolate the NIR emission lines, a near-infrared bandpass filter was employed. (**c**) BT colloidal suspension and BT powder were compared to background noise under argon-NIR laser illumination. Samples of BT were excited using an argon-ion-based laser beam passed through a NIR bandpass filter (700 nm–1100 nm), with potential SHG emission signals recorded in real time using an Ocean Optics QE Pro Spectrometer (Ocean Insight, USA).

Our goal was to identify emission lines around the 400 nm region from our BT samples upon illumination with the accessible and affordable CW Argon laser. To achieve this, we first recorded the emission spectrum of the Argon laser source (Figure 4a). The Argon laser emits multiple NIR lines within a broad range of wavelengths, spanning from 696 nm to 968 nm (see also Supplementary to 3.3.1). Peaks around 700–900 nm seem prominent (Figure 4b), with a cluster of closely spaced lines near 800 nm.

We hypothesize that these closely spaced NIR lines around ~800 nm from the CW Argon-ion laser may overlap temporally within the coherence time, effectively behaving like a single illumination line. Constructive reinforcement of the local electric field—per Equation (2) in Introduction—facilitates SHG under CW conditions. We effectively treat the ~800 nm cluster of closely spaced NIR lines as a single excitation line, since this region shows the strongest overlap with BT defect and SHG-active states. Although an average power of 25 mW was used, the interaction of these limited-power photons with nanoparticles increases the effective intensity (I = P/A), amplifying the local electric field and enhancing nonlinear effects. This increased field strength can amplify the probability of SHG (both directly, by generating emission with complete SHG information, and indirectly, by requiring identical frequencies), with TPA probably also being influenced by the closely spaced frequencies-more flexible than SHG-, albeit with extremely low rates.

Moreover, the simultaneous and continuous interaction of multiple emission photons from the Argon laser with nonlinear BT nanoparticles was expected to generate a shifted SHG wave [28,48]. BT’s magnetic dipole or quadrupole resonances could further influence this SHG emission by affecting the interaction dynamics between the laser photons and the nanoparticles, enhancing the overall SHG response [28] (see Introduction, Section 1).

With this understanding, we measured the emission spectra of BT suspension samples and BT powder, comparing them to the background noise. Figure 4c displays the three non-concentric and shifted emission spectra obtained, suggesting complex interactions in our light–matter system [37,57,77]. The emission intensities followed a trend: I (BT suspension) > I (BT powder) > I (background). This trend was expected, as the powder form induces greater scattering, which diminishes emission intensity, whereas the dispersion of particles in the BT suspension may enhance absorption, resulting in higher emission intensities. The SHG signal obtained in our experiments showed good agreement with the expected SHG efficiency (η) for CW lasers in nanoscale systems based on experimental setup parameters and previously reported studies for CW lasers. Given the known SHG efficiency, typically ranging from 10^−4^ to 10^−8^ per Watt for similar CW systems (~10^−8^ to 10^−4^ W^−1^) vs. pulsed (>10^−2^ W^−1^), and the excitation power P of 25 mW (0.025 W), the observed SHG intensity aligns with the anticipated range after accounting for experimental conditions and intrinsic system losses (I_SHG_ ∝ η⋅P^2^). This consistency strengthens the claim for SHG detection in our system. To further validate this conclusion and gather complementary evidence for SHG-induced ROS generation, we employed a singlet oxygen trap, a DPBF indicator, as discussed in the next section.

#### 3.3.2. Investigation of Nonlinear Optical Processes via ROS Generation in BT Nanoparticles Under NIR Continuous Argon Laser Illumination

To elucidate the dominant mechanisms in our system, involving continuous Argon-ion multiline NIR illumination and BT-based nanoparticle suspensions, we employed DPBF as a singlet oxygen scavenger. The power pump source (average 25 mW) was appropriate for monitoring singlet oxygen production through stable kinetics. In the initial phase of our investigation, we focused on ROS production under NIR illumination of the BT suspension. BT and DPBF were illuminated individually in glass vials, as well as in combination (see Section 2.8). As depicted in Figure A1(B1) in Appendix B, BT and DPBF were combined at absorbance levels of 0.035 and 0.25, respectively, at 410 nm to ensure full singlet oxygen trapping by DPBF and to prevent any aggregation-induced spectral shifts [37,78]. Moreover, ensuring the suitable absorbance levels between the analyte and the DPBF indicator minimizes the effects of spectral overlap (see also the Supplementary to 3.3.2).

**Figure 5:**
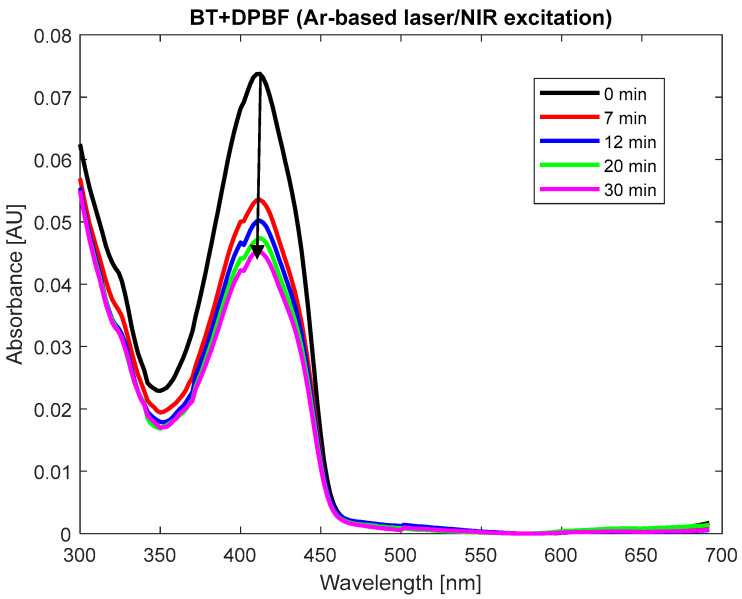
An investigation of the ROS production process by BT (with BT and DPBF absorbance levels of 0.035 and 0.25, respectively): absorption spectra of BT + DPBF mixture under closely spaced multi-line emission from argon-ion-based laser near-infrared illumination (average power density of 25 mW/cm^2^) over time.

**Figure A2:**
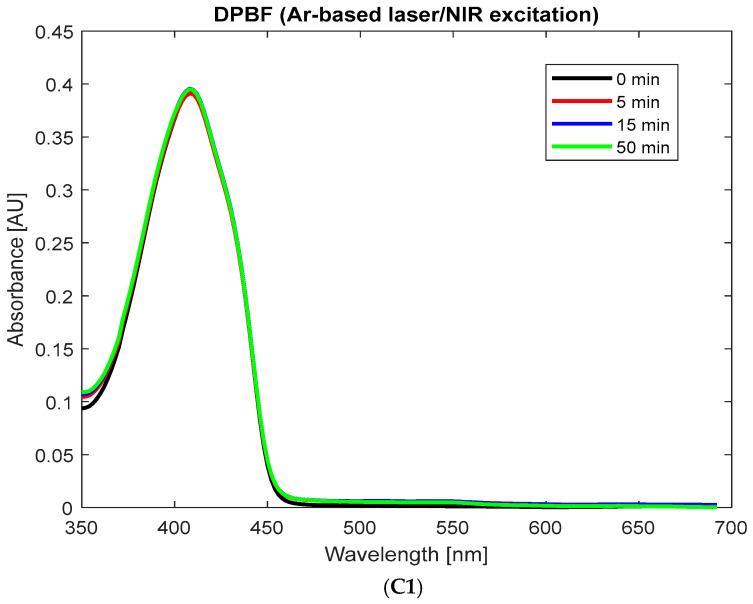

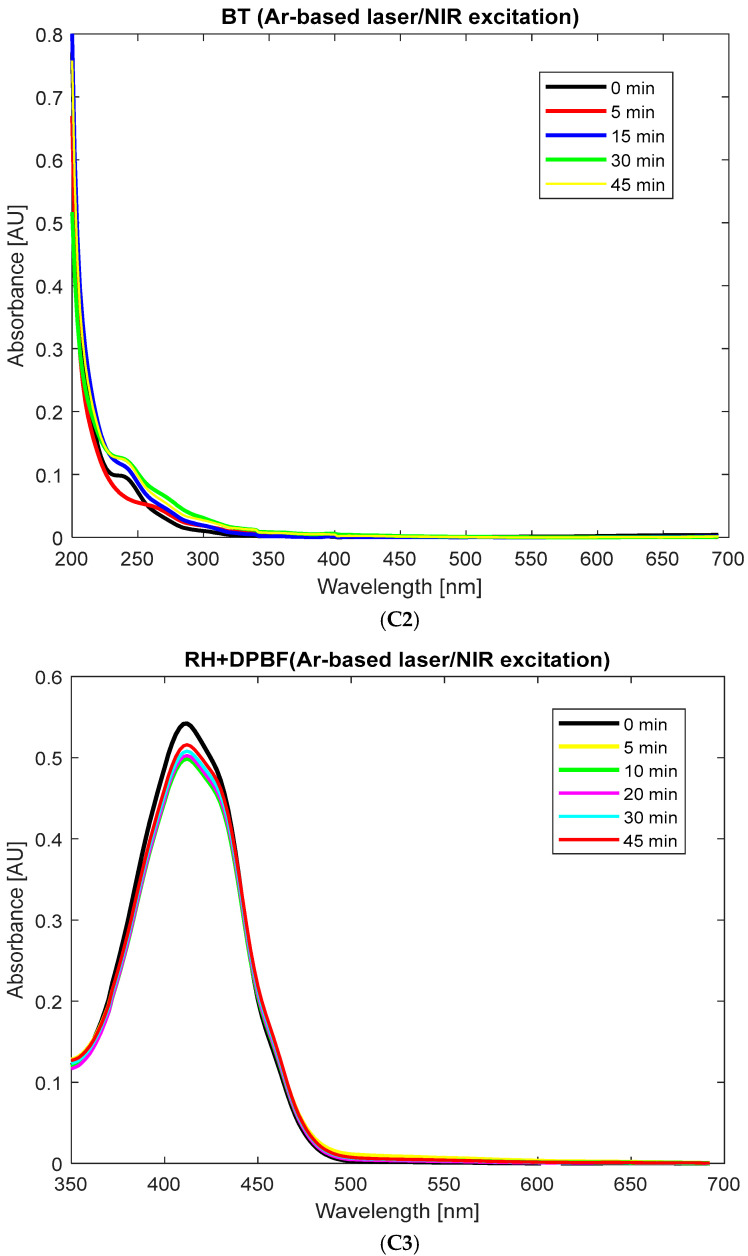
An investigation of the photodegradation processes of DPBF, BT, and rhein + DPBF. The absorption spectra of (**C1**) DPBF alone, (**C2**) BT alone, and (**C3**) rhein (RH) alone under argon-ion-based laser near-infrared illumination (average power density of 25 mW/cm*^2^*) over time. To isolate the closely spaced NIR multi-emission lines for excitation, a near-infrared bandpass filter was employed.

Upon NIR illumination, neither DPBF nor BT displayed significant changes in their absorption spectra (Figure A2(C1,C2) in Appendix C). However, a decrease in the absorption of the BT + DPBF mixture (Figure 5), particularly at 410 nm, DPBF’s maximum absorbance band, was observed, indicating ROS generation (refer to Section 2.8). Notably, BT itself lacks absorption bands between 350 and 450 nm, absorbing mainly in the deep UV region (Figure 2a,b).

Several mechanisms may explain the observed trends: **(a) Electron–Hole Pair Formation:** BT nanoparticles possess a bandgap of approximately 3.6 eV [79,80,81] and photons in the 700–900 nm range carry between 1.72 and 1.55 eV each [79,80,81]. Furthermore, BT nanoparticles often have defect states or trap levels within their bandgap, allowing for the absorption of sub-bandgap photons. Multi-line emission from the laser might excite these defect states, facilitating electron–hole pair formation. Additionally, plasmonic enhancements from BT nanoparticles, surface interactions (as depicted in Figure 1c), or potential energy shifts caused by local excitons or defects could compensate for the remaining energy gap, contributing to the absorption process (through effective excitation pathways) [75,76,77,82,83]. Moreover, the separation of the electron–hole pairs could lead to charge redistribution within the material, generating local electric fields. These fields might stabilize the pairs, increase their lifetime, and enhance their interaction with water and oxygen molecules, thereby facilitating ROS production. **(b) Piezoelectric Effects:** BT nanoparticles could also contribute to ROS production through piezoelectric catalysis, where mechanical forces, such as ultrasound, applied to the piezoelectric material induce catalytic effects via electric polarization, thereby facilitating ROS generation. **(c) Surface Charge Effects:** The surface charges of BT nanoparticles could influence the local electric field and play a role in ROS generation. BT nanoparticles have high refractive indices,(*n*(λ)), that may concentrate the electromagnetic field near their surface, increasing the effective temporal local photon flux [75,76,77,82,83]. **(d) Photothermal Effects**: potentially due to enhanced local electric fields generated by SHG. **(e) DPBF Photodegradation**, or **(f) Impurities** within the system, may induce ROS production under NIR illumination.

The fourth mechanism, involving photothermal effects from BT, is improbable given the limited power of the pump source used. However, localized fields acting as “hot spots” for ROS generation via an indirect SHG pathway remain a possibility. Regarding DPBF photodegradation, Figure A2(C1) Appendix C rules out this possibility by demonstrating the photostability of DPBF under NIR illumination over time, reinforcing the notion that DPBF did not undergo photodegradation. Similarly, Figure A2(C2) in Appendix C highlights the photostability of BT nanoparticles under NIR irradiation. The possibility of ROS production due to impurity excitation seems negligible; if significant, it would have appeared in the absorption and emission spectra of BT samples (Figure 2 and Figure A2(C2) in Appendix C).

Given these factors, we hypothesized that the SHG effect, coupled with TPA (albeit at a very low level), likely contributes to the observed ROS generation, with electron–hole pair formation being a dominant pathway, particularly given that both SHG and TPA are commonly associated with ROS production [38,83]. Additionally, the 50 nm size of the BT nanoparticles (Figure 1), which is close to the wavelength of the incident light (800 nm), may enhance local electric fields through resonant scattering processes (e.g., Mie scattering). This interaction further increases the probability of nonlinear optical effects, amplifying the generation of ROS via electron–hole pair formation and contributing to the observed trends.

BT is recognized for its efficient SHG capabilities compared to other nonlinear optical effects [22,23,26,27,30,38,39]. Therefore, applying a broad spectrum of multi-line wavelengths to BT nanoparticles, *n*(λ), especially in suspension, is justified to increase the probability of nonlinear optical effects. Finally, alongside the results obtained using the DPBF indicator, we aimed to further investigate the presence of the SHG effect in our system by examining its distinctive properties (see the Supplementary to 3.3).

#### 3.3.3. Investigation of Nonlinear Optical Processes via ROS Generation in BT/Rhein Phyto-Nanoconjugates Under NIR Continuous Argon Laser Illumination

Rhein features an extended π-conjugated system of three fused aromatic rings (see Scheme A1), with carbonyl, hydroxyl, and carboxylic acid groups [70,71,72], which may enhance its interaction with BT nanoparticles. Its bandgap (3.2 eV) [84] is comparable to that of BT nanoparticles (3.2–3.6 eV), facilitating efficient energy transfer and enhanced ROS generation. The interaction in our organic–inorganic rhein/BT system induces a dynamic effect upon photoexcitation. Rhein, with higher HOMO-LUMO energy levels than BT’s valence–conduction bands, shares the same bandgap. Upon excitation, rhein may generate excitons, leading to charge transfer and/or hopping from the rhein LUMO to the BT conduction band, resulting in charge separation and polarization. This enhances the nonlinear response, with rhein becoming positively charged and BT negatively charged, amplifying their electrostatic interaction (exciton–plasmon coupling). Given the strong light interaction of π-conjugated molecules and the potential of coatings to improve nonlinear optical properties or prevent agglomeration, we investigated the ROS generation capability of BT/rhein nanoconjugates under NIR illumination. Additionally, the BT/rhein nanoconjugates demonstrated photoactivity in PC3 cancer cells (Section 3.2). Therefore, our investigation focused on assessing their ROS production using the DPBF probe.

In our previous studies [22,37], we investigated ROS production in cells exposed to BT/Phyto-PS nanoconjugates with similar spectral properties (curcumin vs. Rhein, protoporphyrin vs. extract). Fluorescence emission intensity (FEI) readings showed that protoporphyrin required long pre-incubation time (~4 h) for significant accumulation within the cells, while curcumin reached stable levels with short incubation (within 0.5–1 h), see also refs. [21,45] regarding the FEI trend. Moreover, as shown in [37], lower active compound concentrations as in nanoconjugates are likely to demand longer pre-incubation to trigger intracellular ROS. Since we performed short pre-incubation time, ROS is mainly extracellular, consistent with viability data in Figure 3. All together suggest that shorter pre-incubation is sufficient for Rhein nanoconjugates. However, DCFH-DA as an intracellular ROS indicator is unsuitable in this system due to spectral overlap with Rhein’s fluorescence, which would interfere with accurate ROS quantification. While DPBF is commonly used for extracellular ROS detection, it also has some spectral overlap with Rhein. However, given that the observed ROS in this study are primarily extracellular due to the short pre-incubation times, DPBF remains a reliable method for measuring extracellular ROS.

**Figure 6:**
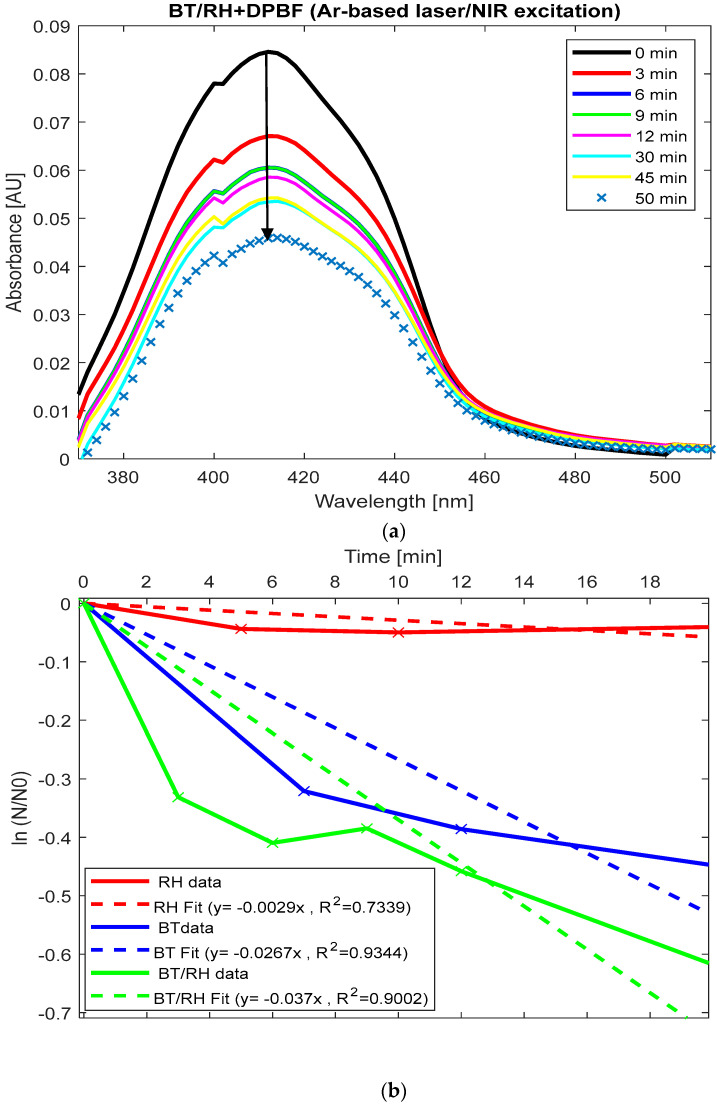
An investigation of the ROS production process by BT/rhein nanoconjugates and their individual components (BT and rhein)**.** (**a**) The absorption spectra of BT/rhein + DPBF samples under closely spaced multi-line NIR illumination from an argon-ion-based laser (averaging 0.25 mW) over time. (**b**) The natural logarithm of the normalized DPBF absorption at 410 nm in the presence of BT, rhein (RH), and BT/rhein (BT/RH) as a function of time, displaying singlet oxygen production rate constants for each case.

A comparative analysis was performed between the BT/rhein nanoconjugates and their individual components (BT and rhein) regarding the singlet oxygen production rate under NIR illumination [85,86,87]. The experiments were conducted using a 25 mW average excitation power source under conditions nearly identical to those used for BT alone (Figure 5). The absorbance intensity levels between BT/rhein and DPBF were 0.035 and 0.25, respectively, and those between rhein and DPBF were 0.25 and 0.4, respectively, at 410 nm (Figure A1(B2,B3) in Appendix B). Figure 6a displays the absorption spectra of (BT/rhein) + DPBF upon NIR illumination over time, showing a reduction under our experimental conditions. The absorbance decreases sharply from 0.087 at 410 nm (time zero) to 0.057 at 30 min, indicating significant ROS generation during this period. After 30 min, the absorbance fluctuates slightly (e.g., rising to 0.059 at 45 min), suggesting that ROS generation may plateau or the reaction rate decreases over time. By normalizing the absorbance of each sample at 410 nm (derived from Figure 5 and Figure 6a) to the initial concentration at time zero, applying the natural logarithm to the resulting values, and subsequently plotting these values as a function of time, we observed the trend of singlet oxygen production rate constants (k) (Section 2.8). This was obtained from the slope of the linear fit for each case (Figure 6b). Beyond these time periods, the reaction transitioned to alternative pathways.

For long illumination times (20–30 min), the rate constants for ROS generation in BT and BT/rhein nanoconjugates are comparable (k(BT) = 0.026 ± 0.0072 min^−1^ vs. k(BT/rhein) = 0.037 ± 0.0354 min^−1^, *p*-value > 0.05); see Figure 6b. However, for shorter illumination times (up to 7 min), the decrease in absorbance is more pronounced for the BT/rhein nanoconjugates, as shown by the change from 0.087 to 0.059 in the first 7 min compared to the reduction from 0.073 to 0.053 in the BT case (Figure 5 and Figure 6a,b). The addition of rhein to BT enhanced ROS production at short illumination times, as evidenced by this absorbance change (Figure 6a,b). This suggests that the addition of rhein enhances the initial ROS production rate in the early stages, but over longer times, the difference between the two systems becomes less significant. Rhein’s photosensitizing properties in the visible spectrum are known [47], and in our BT/rhein nanoconjugated system, the involvement of the SHG effect may contribute to ROS production, which is subsequently trapped by the DPBF indicator. On the other hand, rhein alone (rhein + DPBF) did not exhibit any photo-response under direct NIR illumination, meaning no ROS production was observed (Figure 6b and Figure A2(C3) in Appendix C). This observation suggests that, under our experimental conditions (including the laser source’s wavelength and power, incident light polarization, sample concentration, and irradiation time), rhein alone cannot participate in SHG processes.

The comparison between the three cases is summarized as follows: k(rhein) = 0.0029 ± 0.0021 min^−1^ < k(BT) = 0.0267 ± 0.0072 min^−1^ < k(BT/rhein) = 0.037 ± 0.0354 min^−1^ (Figure 6b). Since rhein acts as a PS-responsive to blue-visible light with a maximum absorbance at 433 nm, it can be excited by SHG emission from BT, leading to ROS production [22,23,24,25,26,27,28,29,30,31,32]. Subsequently, its emission, within the range of 450–750 nm (Figure 2d), facilitates energy transfer to the BT, thereby enhancing ROS generation. Spectral overlap between DPBF and rhein may overestimate ROS generation in BT/rhein, exaggerating differences from BT.

#### 3.3.4. Nonlinear Optical Interactions of BT/rhein and BT/EXT Nanoconjugates with High-Power Femtosecond Pulse Laser Illumination

In the next phase of our study, we compared the results obtained using the CW Argon-ion laser with those from a high-peak-power, 800 nm femtosecond pulsed laser (Section 2.9 and Section 2.10).

**Figure 7:**
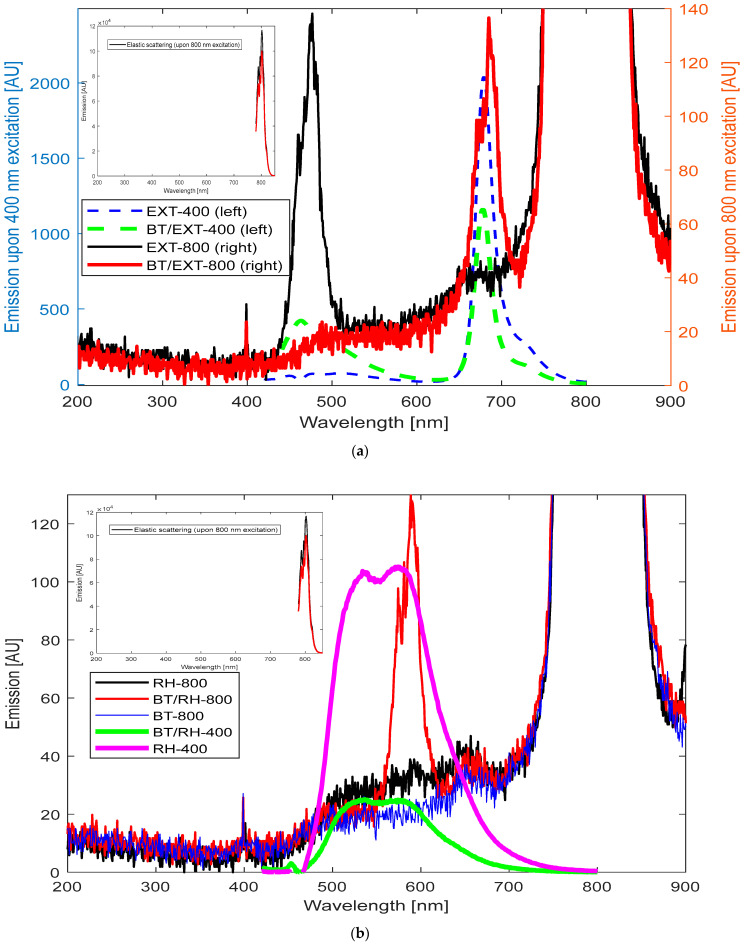
A comparison of high-peak-power femtosecond pulse laser excitation at 800 nm with 400 nm blue light excitation for the following samples: (**a**) extract (EXT) alone and BT/EXT/PEG100 nanoconjugates; (**b**) rhein (RH) alone, BT/rhein/PEG100 nanoconjugates, and BT alone. The samples were excited using a high-peak-power femtosecond pulse laser (emission wavelength of 800 nm, average power density of 300 mW/cm^2^, pulse length of 35 fs, and repetition frequency of 500 Hz), with emission signals recorded in real time using an Ocean Optics QE Pro Spectrometer (Ocean Insight, USA). The inset subfigures in (**a**,**b**) show the emission profile of the femtosecond laser source used for sample illumination.

We examined samples of (a) EXT, (b) rhein, (c) BT, (d) BT/EXT/PEG100, and (e) BT/rhein/PEG100, subjecting each to high-intensity 800 nm femtosecond pulse laser illumination. We then compared their emission spectra under 800 and 400 nm illumination (Figure 7a,b). This approach allowed for a comprehensive analysis of potential nonlinear optical effects and their differentiation.

As demonstrated, in the case of BT/EXT nanoconjugates under 800 nm illumination, the overall emission spectra displayed a similar pattern to that observed with 400 nm excitation. Specifically, the BT/EXT-800 sample under 800 nm illumination exhibited emission signals at 488 nm and 684 nm, corresponding to the individual BT and EXT emission signals, respectively, mirroring the overall shape of the spectra obtained under 400 nm excitation (Figure 7a). This result emphasizes that the transition to a higher power pulse laser source induced a TPA nonlinear effect. In TPA, two photons (in this case, at 800 nm) are absorbed simultaneously, leading to the excitation of a molecule to an electronic state that could also be reached via single-photon excitation at 400 nm [82,83]. This implies that, ideally, the dynamics of the relaxation pathway back to the ground state via emission wavelength distribution should be the same for both 400 nm and 800 nm excitation. In our case, a slight red shift of 4 nm was observed for 800 nm excitation compared to 400 nm excitation. This red shift suggests that while TPA is occurring, the emission process may be slightly influenced by local environmental factors, solvent effects, reabsorption, or population differences. Additionally, comparing the emissions of BT/EXT-800 with those of EXT-800 and EXT-400 reveals that upon 800 nm illumination, the nanoconjugates produced an emission signal at 684 nm. This behavior reflects the direct excitation of EXT at 400 nm, as the emission signal was absent in the case of EXT-800 alone. These results strongly support the presence of the SHG effect under our light–matter conditions [88,89,90]. It should be noted that for the extract alone, excitation at 800 nm produced an emission maximum at 476 nm, which is consistent with a potential TPA effect. This emission corresponds to the extract’s absorption maximum wavelength (Figure 2a), further supporting the probability of TPA. The BT/rhein nanoconjugate’s emission spectra under 800 nm illumination were more complex to analyze (Figure 7b). The spectra revealed a sharper distribution in the range of 560 to 630 nm, with three maxima at 574, 581, and 588 nm. These maxima overlap with the lower energy band (with a maximum at 588 nm) seen at 400 nm excitation. This suggests that at 800 nm illumination, three closely spaced energy levels are involved in the emission process compared to a single broad energy band observed at 400 nm excitation. The broad energy band between 460 nm and 560 nm seen at 400 nm excitation was absent at 800 nm illumination. Additionally, comparing the emissions of BT/RH-800 with those of RH-800 and RH-400 reveals that upon 800 nm illumination, the nanoconjugates produced an emission signal in the range of 560 nm to 630 nm. This behavior resembles the direct excitation of RH at 400 nm, as the emission signal was absent in the case of RH-800 alone. These results strongly support the presence of the SHG effect in the system. The polarization induced by the carbonyl functional group in rhein (an anthraquinone) enables charge separation, with delocalized electrons contributing to its nonlinear optical behavior.

In conclusion, the BT/rhein nanoconjugates demonstrated the simultaneous absorption of two photons, leading to excitation and subsequent relaxation from lower-energy states. This observation strongly indicates the involvement of the TPA effect in the process. The emission spectra of BT/RH-800 compared to those of RH-800 and RH-400 suggest the role of SHG. Furthermore, the sharpness of the BT/RH’s emission spectra underlines the presence of nonlinear optical effects (selective transitions) compared to the broader emissions typically associated with linear excitation processes.

Similarly, in both the BT/rhein and BT/extract nanoconjugates, we observed comparable spectral features under blue and NIR illuminations, indicating the presence of SHG. This resulted in blue light emissions that produced photochemical effects comparable to those from direct blue light excitation. Additionally, BT under 800 nm illumination showed a small and monochromatic signal at 400 nm, consistent with the SHG effect, while rhein alone under 800 nm illumination did not exhibit any excitation/fluorescence transitions.

For the BT/extract nanoconjugates, utilizing a high-power femtosecond pulsed laser for excitation can enhance nonlinear optical performance. In the case of BT/rhein, a complex light–matter interaction was observed, involving multiple nonlinear optical effects during the excitation–emission process.

## 4. Conclusions

We successfully demonstrated that the interaction between a CW Argon-ionlaser with closely spaced multiple emission lines in the NIR region and the BT/Phyto-PS nanoconjugates induces nonlinear optical effects, leading to the generation of ROS and a decrease in cell survival. Additionally, our investigation demonstrated that the BT/rhein nanoconjugates were more versatile, effectively mediating a combined NIR-induced SHG-PDT treatment, while the BT/extract nanoconjugates showed efficacy primarily in the dark. The nanoconjugates were synthesized using a low-frequency probe sonication method, which enabled the effective conjugation of barium titanate nanoparticles with the PSs, facilitating their structural integrity and functionality. The SHG signal observed in our experiments corresponded closely with theoretical predictions and prior findings for CW lasers. This was further corroborated through validation with the ROS indicator DPBF, which demonstrated a reduction in absorbance at 410 nm under NIR illumination, indirectly affirming SHG emission at 400 nm from BT/rhein nanoconjugates. Using both CW and pulsed lasers, we also established a direct and real-time method for quantifying ROS generation through SHG signals, offering a new pathway for ROS monitoring. The use of a high-power femtosecond pulsed laser further enhanced the nonlinear optical performance of the PSs and their nanoconjugates, particularly that of the BT/extract. The extract alone exhibited a measurable TPA effect, highlighting its intrinsic nonlinear optical properties independent of BT nanoparticles. This observation may suggest the structure of the predominant molecular phase in the extract, warranting further exploration in nonlinear optics. The BT/rhein nanoconjugate displayed complex light–matter interactions, suggesting the involvement of multiple nonlinear optical effects. Both BT/extract and BT/rhein can be used for SHG/TPA-based treatments. Future work may include long-term in vivo validation.

## Data Availability

The data supporting the results of this study are available in the Supplementary File. All relevant datasets are included in the Supplementary File and can be accessed by readers. Any additional data or information will be available upon request from the corresponding author.

## Supplementary Materials

The following supporting information can be downloaded at https://www.mdpi.com/article/10.3390/polym17131831/s1, Figure S1: The effect of fundamental wave polarization on BT’s capacity for ROS generation under argon-ion-based NIR illumination (averaging 0.375 mW) over time. (a) The natural logarithm of the normalized DPBF absorption at 410 nm in the presence of BT, showing singlet oxygen production rate constants for each polarization angle case (0°, 30°, and 70°) as a function of illumination time. (b) Dependence of BT’s singlet oxygen production rate constants, k (min^−1^) on average excitation power. The singlet oxygen production rate constants, k, of BT + DPBF samples exposed to different NIR illumination powers (0, 0.037 mW, and 25 mW) were investigated and plotted as a function of the incident beam power and analyzed for trends. Figure S2A: The emission spectrum of an argon-ion-based laser features simultaneous and continuous spectral lines in the near-infrared region (696 nm to 922 nm) after passing through a near-infrared bandpass filter. This region includes Ar+ 2p→1s transitions, with closely spaced multi-emission lines providing an average power density of 0.375 mW/cm2. Figure S2B: The effect of fundamental wave polarization on BT’s capacity for ROS generation under argon-ion-based NIR illumination (averaging 0.375 mW). Normalized absorption spectra of BT + DPBF samples under NIR illumination after passing through polarizers set at (SB1) 0°, (SB2) 30°, and (SB3) 70°, relative to the p-polarized fundamental wave, over time. Table S1: BT’s singlet oxygen production rate constant varying with average power excitation.

## Appendix A

Appendix A includes Scheme A1, Scheme A2, Scheme A3, Scheme A4 and Scheme A5, which illustrate the experimental setup for NIR-induced SHG-PDT applied to PC3 cancer cells and SHG emission signal detection in various configurations. Additionally, Scheme A1 and Scheme A5 depict the sonodynamic mechanism’s role in enhancing ROS production during nanoconjugate synthesis through sono-mechanical and sonochemical effects.

## Appendix B

Appendix B includes the absorbance levels of (**B1**) BT, DPBF, and BT+DPBF, (**B2**) BT/RH nanoconjugates, DPBF, and BT/RH+DPBF, (**B3**) RH, DPBF, and RH+DPBF at 410 nm at time zero (before illumination).

## Appendix C

Appendix C includes a photodegradation investigation of (**C1**) DPBF, (**C2**) BT, and (**C3**) RH+DPBF, serving as controls, under NIR argon-ion-based illumination over time.
