# Supplementary material for: Near-Infrared and Sono-Enhanced Photodynamic Therapy of Prostate Cancer Cells Using Phyto-Second Harmonic Generation Nanoconjugates
Source: Polymers (Basel). 2025 Jun 30;17(13):1831. doi: 10.3390/polym17131831 (PMC12251607; doi:10.3390/polym17131831)
Supplement: Supplementary file 1 [file polymers-17-01831-s001.zip › polymers-3694909-supplementary.pdf]

Supplementary to 3.3.1.

Argon is a naturally abundant, inert noble gas that involves ionization processes [48-51]. Additionally, argon exhibits low thermal conductivity, which reduces the probability of internal conversion and vibrational relaxation pathways [37,48-51]. That means that the fluorescence relaxation pathways leading back to the ground state are more probable. The discrete energy transitions responsible level for laser action in argon originate from its ionized states, resulting in powerful emissions achieved through a two-stage process including ionization followed by excitation (NIR includes Ar^{+} 2p→1s transitions) [48-51]. Argon lasers provide multiple emission wavelengths, with the most intense emissions in the blue and green regions of the visible spectrum, along with secondary emission lines in the NIR range [48-51]. These emissions encompass 26 distinct wavelengths (696 nm, 706 nm, 726 nm, 737 nm, 751 nm, 762 nm, 771 nm, 788 nm, 790 nm, 794 nm, 800 nm, 810 nm, 826 nm, 841 nm, 851 nm, 866 nm, 877 nm, 890 nm, 912 nm, 922 nm, 937 nm, 939 nm, 960 nm, 965 nm, 966 nm, and 968 nm), where the most relevant for the effective excitation of BT nanoparticles are those within the range of 800 nm to 1400 nm [26-28]. Among the emission lines produced by the argon laser, 16 wavelengths fall within this optimal range, with the most notable contributions observed at 800 nm and 810 nm (averaging 25 mW- Fig.4(b)).

Supplementary to 3.3.2.

The primary medium for the colloidal BT nanoparticles analyte is DDW, which is significant to take into account in terms of absorbance intensity considerations. Conversely, DPBF is a yellow dye known for its notable absorbance, with methanol serving as a "good solvent" due to its ability to facilitate its dissolution as well as to enhance the lifetime of ROS. Literature supports the choice of an absorbance level of 0.25 for the singlet oxygen indicator, as this directly correlated concentration ensures that the indicator is sufficiently available or in access to facilitate complete singlet oxygen trapping while also preventing any aggregation bands [37,82]. Furthermore, maintaining appropriate absorbance levels between the analyte and the DPBF indicator is crucial for minimizing the effects of spectral overlap. In our experimental setup, we utilized 2 ml of the BT nanoparticles sample in DDW (with an absorbance intensity of 0.035) and 1 ml of the DPBF indicator in methanol (with an absorbance intensity of 0.25), resulting in a total sample volume of 3 ml (see Section 2.8). These considerations collectively influenced our selection of the absorbance levels for the nanoparticle's suspension analyte and the DPBF indicator.

The primary surface hydroxyl groups on $BaTiO_3$, namely Ti-OH and Ba-OH, which are linked to titanium and barium atoms on the surface, respectively, can be generated through interactions with water molecules. Depending on the pH of the surrounding medium, or even due to light treatments, these hydroxyl groups can

undergo protonation or deprotonation, influencing the surface charge and potentially contributing to ROS production.

Supplementary analyses to 3.3: Polarization and Power Dependence in SHG-Driven Nanoparticle Systems

To further investigate the operational mechanisms in our system involving continuous argon-ion-based, closely spaced multi-photon NIR fundamental frequencies interacting with BT nanoparticles- and BT-based phyto-nanoconjugates, we conducted polarization and power dependence experiments. Although the system consists of nanoparticles in suspension, polarization dependence is a distinctive characteristic of SHG phenomena [26-27], as induced anisotropies and local field effects can lead to measurable responses. The BT+DPBF samples were illuminated with argon-NIR light after passing through polarizers set at 30°, 70°, and with no polarizer used as a control (see Section 2.11). It is worthwhile to mention that the original fundamental pump source exhibited P-type polarization. Additionally, to investigate power dependence, the radiation intensity was reduced to one-fifth of that used in the previous experiments (Fig. 4b vs. Figure S2A in Supplementary A).

Dynamic symmetries in the driving laser field can establish selection rules governing harmonic emissions. Specifically, a linearly polarized driving laser imposes linear polarization on the emitted harmonic lines, as the polarization of the emitted harmonics is determined by the electron’s vector momentum upon impact. In nonlinear optical processes, especially when generating high harmonics in atomic or molecular systems, each harmonic photon can theoretically represent a superposition of frequencies, forming a frequency comb. This suggests that each photon may encapsulate the entire spectrum of the SHG process. In this "photon comb" concept, a single photonic state may carry quantum characteristics, including possible entanglement between the harmonic emission and the driving laser emitting source, and encapsulate information about the entire harmonic process, including details of the energy distribution and cut-off frequency. Additionally, the *sinc* envelope around or inherent to the laser's fundamental frequency can preferentially promote harmonic processes close to the fundamental energy and scare the "far" ones.

Considering multiscale dynamic symmetry within the dipole approximation, where each nanoparticle within the BT colloidal suspension is considered as a dipole moment, the driving electric field can induce partial alignment of the nonlinear high polarizability dipole orientation in the suspension (breakage of symmetry), allowing for more coherent build-up of harmonics and stronger nonlinear mixing, which may result in increased ROS generation. This alignment isn’t strict but sufficient to create a net anisotropy in the effective response of the colloidal suspension. This alignment increases the overlap of the laser field’s polarization with the effective birefringence of the colloidal particles (aligns more effectively with the intrinsic optical axis of the birefringent medium), so that the dynamical symmetry of the driving fields and the

intrinsic birefringence of BT nanoparticles can introduce effective anisotropies. These anisotropies allow for phase matching that varies with polarization angle: as the polarization angle of the fundamental source increases, it can lead to better alignment with the dipole moments of the nanoparticles, resulting in stronger constructive interference and improved phase matching. In nanoscale systems, local field effects can generate a pseudo-structured environment, even in a random suspension [23,26-28,36-38, see also Figure 1(c)]. The nonlinear properties of BT nanoparticles enable them to amplify specific field components, thereby affecting and interacting with each other's local fields.

This interaction is particularly pronounced when the external laser field is aligned at an angle that optimizes field coupling between particles.

The kinetic curves presented in Figure S1(a) (derived from Figure S2B; Figures SB(1)-SB(3) in Supplementary B, see also Section 2.8) suggest BT suspension's capacity to generate ROS under our experimental conditions and display the singlet oxygen production rate constants trend for each polarizer configuration: $k_{30} = 0.0274 \pm 0.0083$ min^{-1}, $k_0 = 0.0414 \pm 0.0108$ min^{-1}, and $k_{70} = 0.0398 \pm 0.0157$ min^{-1}. The difference between k_0 and k_{30} was not statistically significant (p-value > 0.05), and the difference between k_0 and k_{70} also showed no statistical significance (p-value > 0.05), indicating that changes in fundamental wave polarization primarily influence ROS generation based on temporal configurations. It should be mentioned that these kinetic rates were derived from a pseudo-first-order kinetic reaction model, as appropriate for our analysis (Section 2.8).

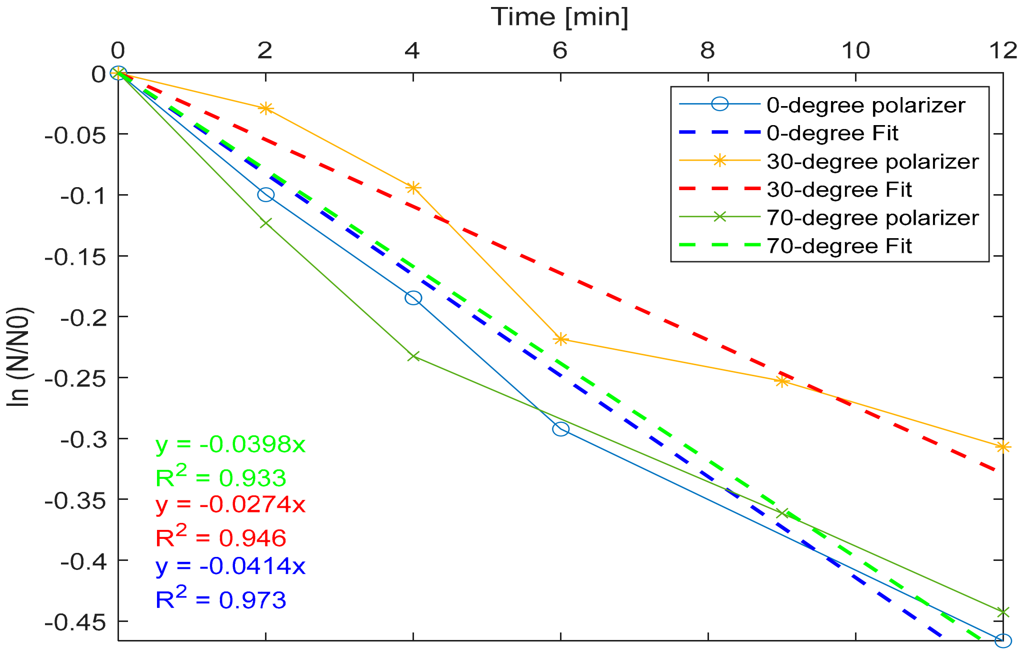

(a)

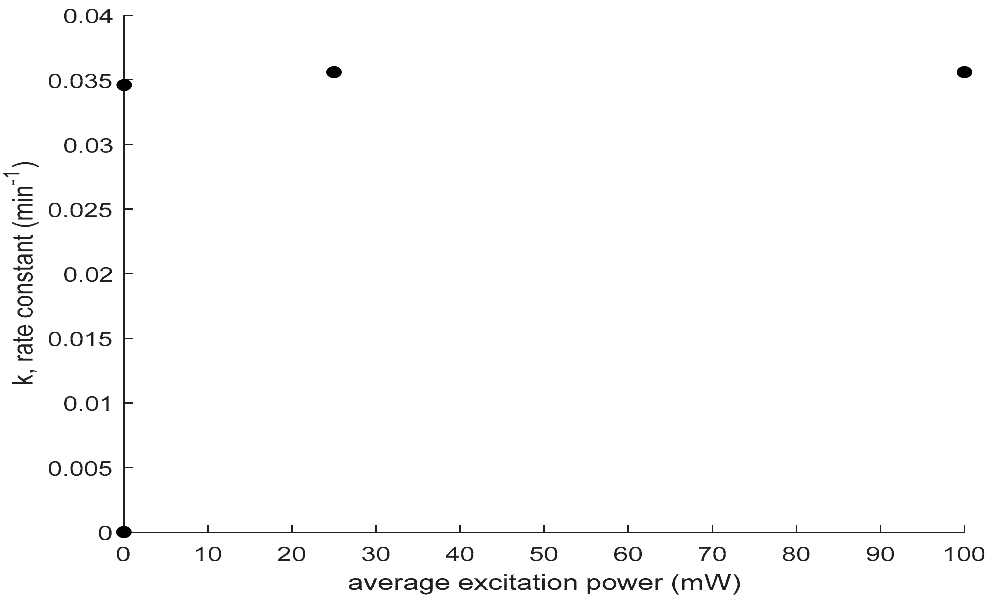

(b)

Figure S1. The effect of fundamental wave polarization on BT's capacity for ROS generation under argon-ion-based NIR illumination (averaging 0.375 mW) over time. **(a)** The natural logarithm of the normalized DPBF absorption at 410 nm in the presence of BT, showing singlet oxygen production rate constants for each polarization angle case (0°,30°,and 70°), as a function of illumination time. **(b)** Dependence of BT's singlet oxygen production rate constants, k (min^{-1}), on average excitation power. The singlet oxygen production rate constants, k, of BT+DPBF samples exposed to different NIR illumination powers (0, 0.037 mW, and 25 mW) were investigated, and plotted as a function of the incident beam power and analyzed for trends.

Taking the singlet oxygen production rate constants, k (min^{-1}), of BT+DPBF samples (Figure 5 VS. 6(b) VS. S1(a)) as a function of the average excitation power, resulted in a non-linear rising trend (see Table S1 and Figure S1(b)). This trend indicates a non-linear power dependence, consistent with findings from previous studies [26-27]. The trend is probably generated by the influence of multiphoton interactions, exhibiting nonlinear mechanisms that support the overall presence of nonlinear optical effects. It should be noted that within the boundaries of the graph, specifically between the starting point at zero intensity (the origin) and infinite intensity (the final point on the graph related to 100 mW), a nonlinear trend was observed. Regardless of the potential behavior at infinity—whether a plateau, an increase, or a decrease (though less likely)—the overall trend remains nonlinear.

The kinetics of ROS production may also serve to distinguish between different non-linear optical effects. For instance, the SHG process is nearly instantaneous, meaning it occurs almost simultaneously as the fundamental wave traverses the nonlinear medium. Consequently, the SHG signal rises and falls in sync with

fluctuations in the fundamental wave's intensity. This precise timing helps to differentiate the SHG signal from other possible emissions, such as fluorescence, which typically has a longer lifetime and would not exhibit such close temporal correlation with the fundamental wave.

In summary, as evidenced by Figures 5 VS. 6(b VS. S1(a)), increasing the fundamental wave power and/or aligning the fundamental wave with the harmonic nanostructure orientation (Figures S1(a) and S1(b)) enhances the direct correlation and synchronization with the emitted wave, thereby amplifying the SHG effect.

Table S1. BT's singlet oxygen production rate constant varying with average power excitation.

**K (BT+DPBF), Singlet oxygen production rate constant [min^{-1}]**	**Average excitation intensity (AU)**	**Average excitation power (mW)**
0	0	0
0.0356	200,000	25
0.0346	10,000	0.375
$\geq$ 25 mW case	800,000	100 (∞)

Supplementary A

Supplementary A includes Figure **S2A** that displayes the NIR closely spaced multi-line emission from the argon-ion-based laser used for excitation (average power density of 0.375 mW/cm^2).

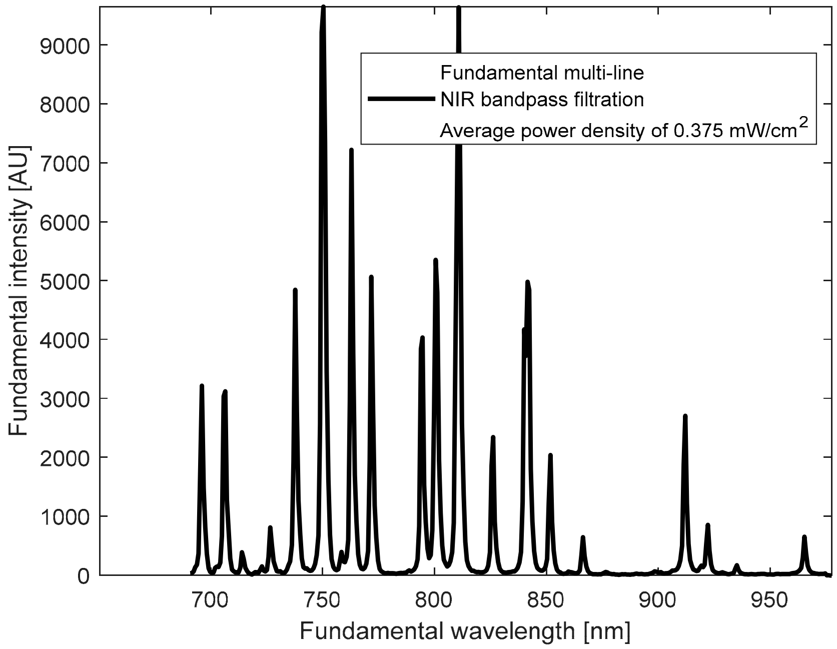

(S2A)

Figure S2A. The emission spectrum of an argon-ion-based laser features simultaneous and continuous spectral lines in the near-infrared region (696 nm to 922 nm) after passing through

a near-infrared bandpass filter. This region includes Ar^{+} 2p→1s transitions, with closely spaced multi-emission lines providing an average power density of 0.375 mW/cm^2.

Supplementary B

Supplementary B includes an investigation of ROS generation in BT samples under argon-ion-based, closely spaced, multi-line NIR excitation over time, with the light passing through polarizers set at 0°, 30°, and 70°.

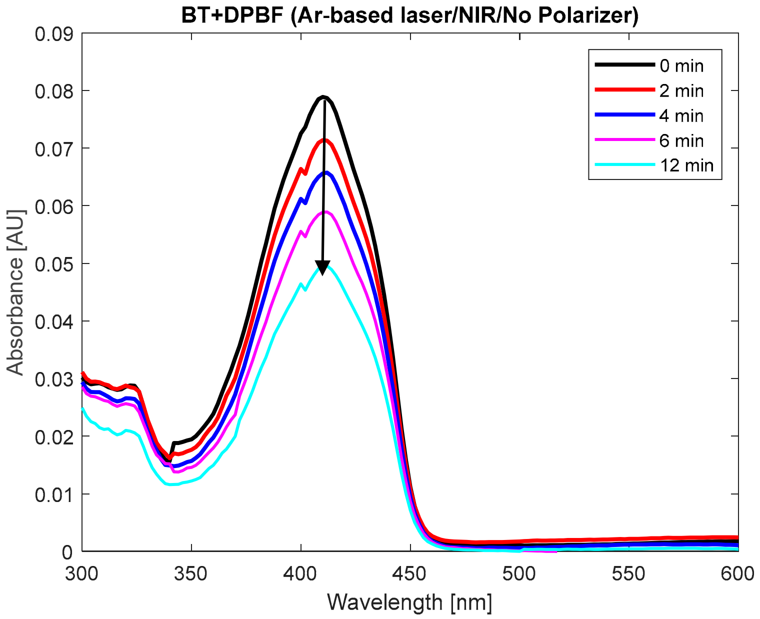

(SB1)

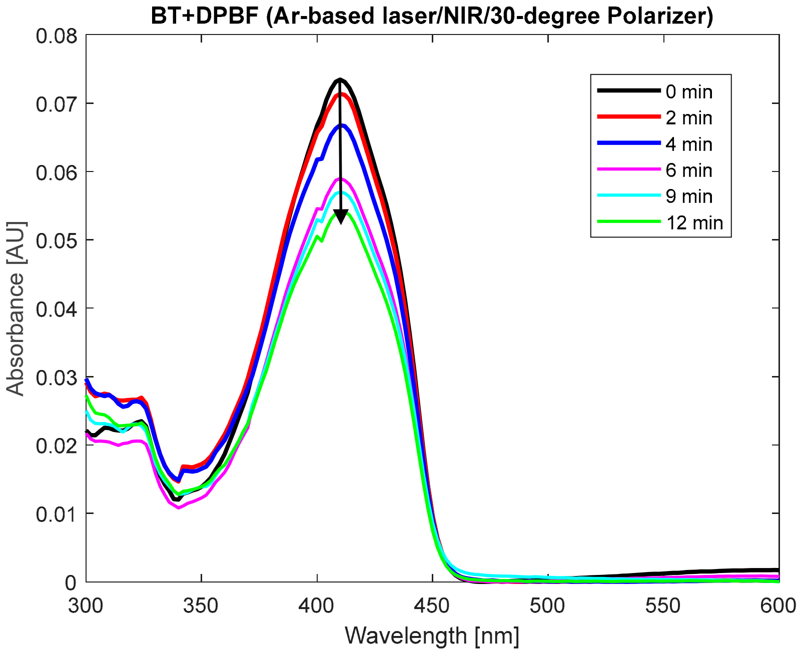

(SB2)

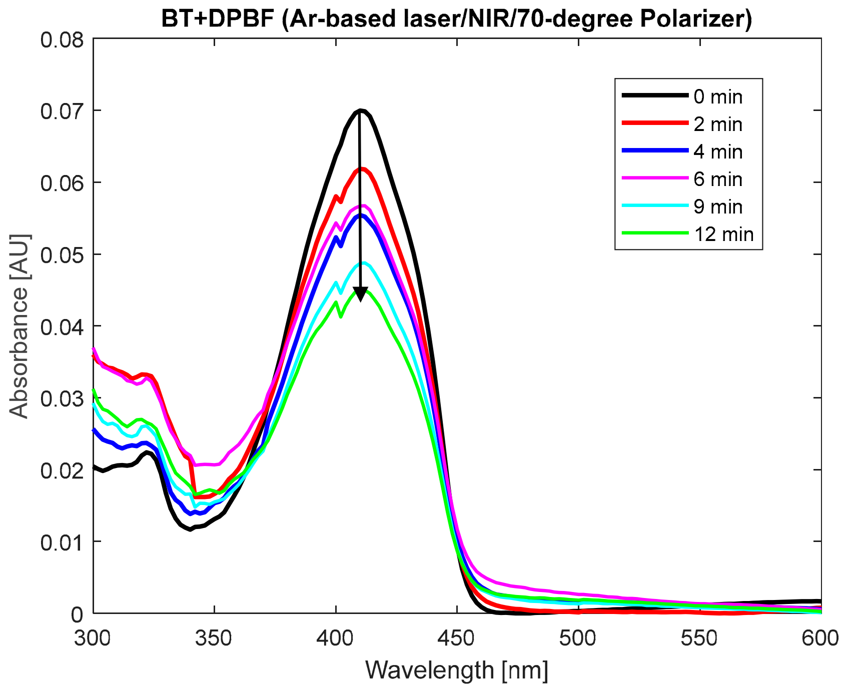

(SB3)

Figure S2B. The effect of fundamental wave polarization on BT's capacity for ROS generation under argon-ion-based NIR illumination (averaging 0.375 mW). Normalized absorption spectra of BT+DPBF samples under NIR illumination after passing through polarizers set at **(SB1)** 0°, **(SB2)** 30°, and **(SB3)** 70°, relative to the p-polarized fundamental wave, over time.
